# Synthesis and characterisation of thiobarbituric acid enamine derivatives, and evaluation of their α-glucosidase inhibitory and anti-glycation activity

**DOI:** 10.1080/14756366.2020.1737045

**Published:** 2020-03-11

**Authors:** M. Ali, Assem Barakat, Ayman El-Faham, Hessa H. Al-Rasheed, Kholoud Dahlous, Abdullah Mohammed Al-Majid, Anamika Sharma, Sammer Yousuf, Mehar Sanam, Zaheer Ul-Haq, M. Iqbal Choudhary, Beatriz G. de la Torre, Fernando Albericio

**Affiliations:** a Department of Chemistry, College of Science, King Saud University, Riyadh, Saudi Arabia; b Department of Chemistry, Faculty of Science, Alexandria University, Ibrahimia, Egypt; c Peptide Science Laboratory, School of Chemistry and Physics, University of KwaZulu-Natal, Durban, South Africa; d KwaZulu-Natal Research Innovation and Sequencing Platform (KRISP), School of Laboratory Medicine and Medical Sciences, College of Health Sciences, University of KwaZulu-Natal, Durban, South Africa; e H.E.J. Research Institute of Chemistry, International Center for Chemical and Biological Sciences, University of Karachi, Karachi, Pakistan; f Dr. Panjwani Center for Molecular medicine and Drug Research, International Center for Chemical and Biological Sciences, University of Karachi, Karachi, Pakistan; g CIBER-BBN, Networking Centre on Bioengineering, Biomaterials and Nanomedicine, and Department of Organic Chemistry, University of Barcelona, Barcelona, Spain

**Keywords:** Thiopyrimidine trione, α-glucosidase inhibitor, antiglycation, molecular docking

## Abstract

A new series of thiobarbituric (thiopyrimidine trione) enamine derivatives and its analogues barbituric acid derivatives was synthesised, characterised, and screen for *in vitro* evaluation of α-glucosidase enzyme inhibition and anti-glycation activity. This series of compounds were found to inhibit α-glucosidase activity in a reversible mixed-type manner with IC_50_ between 264.07 ± 1.87 and 448.63 ± 2.46 µM. Molecular docking studies indicated that compounds of **3g**, **3i**, **3j**, and **5** are located close to the active site of α-glucosidase, which may cover the active pocket, thereby inhibiting the binding of the substrate to the enzyme. Thiopyrimidine trione derivatives also inhibited the generation of advanced glycation end-products (AGEs), which cause long-term complications in diabetes. While, compounds **3a–k**, **5**, and **6** showed significant to moderate anti-glycation activity (IC_50_ = 31.5 ± 0.81 to 554.76 ± 9.1 µM).

## 1. Introduction

Diabetes mellitus (DM) is a disease which caused by a breakdown of carbohydrate metabolism, which plays a significant role in the development of long-term diabetic complications. According to the International Diabetes Federation, 693 million people will suffer from this condition by 2045^1^^,^^2^. DM can be categorised into three types: Type I (T1DM); Type II (T2DM); and gestational (GDM). About 80 − 90% of all DM patients are Type II (T2DM). Drug treatments of T2DM aim to decrease hepatic glucose production, enhance insulin action, and boost insulin secretion from β-pancreatic cells, or block α-glycosidase enzyme (carbohydrate digestive enzymes)^3–6^. Therapeutic in individuals with this disease may lead to various complications, including kidney disease, disorders of the nervous system, leg amputation, heart disease and severe retinopathy up to blindness^7^.

Carbohydrate digestive enzymes are found in the brush border of the intestine. They catalyse the breaking down long-chain polysaccharides into absorbable monosaccharide units. Of these enzymes, α-glucosidases, which play a key role in the digestion and absorption of complex carbohydrates, and has emerged as target to maintain postprandial blood glucose control. α-Glucosidase inhibitors currently used to treat T2DM include acarbose (Precose), voglibose, and miglitol^8^. However, these drugs are associated with several side effects, such as flatulence, stomach-ache, diarrhoea, and liver damage^9^. Therefore, an increasing interest in exploring new drug candidates for glycosidase inhibition is needed^10–12^.

Barbituric acid (BA) derivatives have been reported to have potential anti-hypertensive^13^, anti-cancer^14^, anti-convulsant^15^, anti-inflammatory^16^, anti-psychotic^17^, and antitumor properties^18–21^. Recently, these derivatives have also been reported as anti-diabetic agents^22^. On the other hand, thiobarbituric acid (TBA) analogues has been described to exert anti-inflammatory^16^^,^^23^, immunotropic^24^, anticonvulsant^25^, and anti-hypnotic^25^^,^^26^, anti-neoplastic^27^, and antitumor activities^28^. De Belin et al.^29^ reported a number of TBA derivatives as inhibitors of hypoxia-inducible factor 1 (HIF-1). Recently, Barakat et al.^30^ described the synthesis of a new series of diethylammonium salts of aryl substituted TBA derivatives as α-glycosidase inhibitors. Therefore, given the relevance of TBA derivatives in medicinal chemistry, the design of new molecules containing the thiobarbituric moiety is an inspiring goal.

In continuation of our studies on the synthesis of biologically active compounds^22^^,^^30^^,^^31^, herein, we synthesised 1,3-diethylthiobarbiturate enamine derivatives and evaluated their *in vitro* α-glucosidase inhibitory and anti-glycation activities. In addition, molecular docking studies were performed to study the interactions of the compounds with the catalytic site of the enzyme using acarbose and evaluated their α-glucosidase inhibition capacity and the anti-glycation properties.

## 2. Results and discussion

### 2.1. Synthesis of the target compounds

**Scheme 1.**
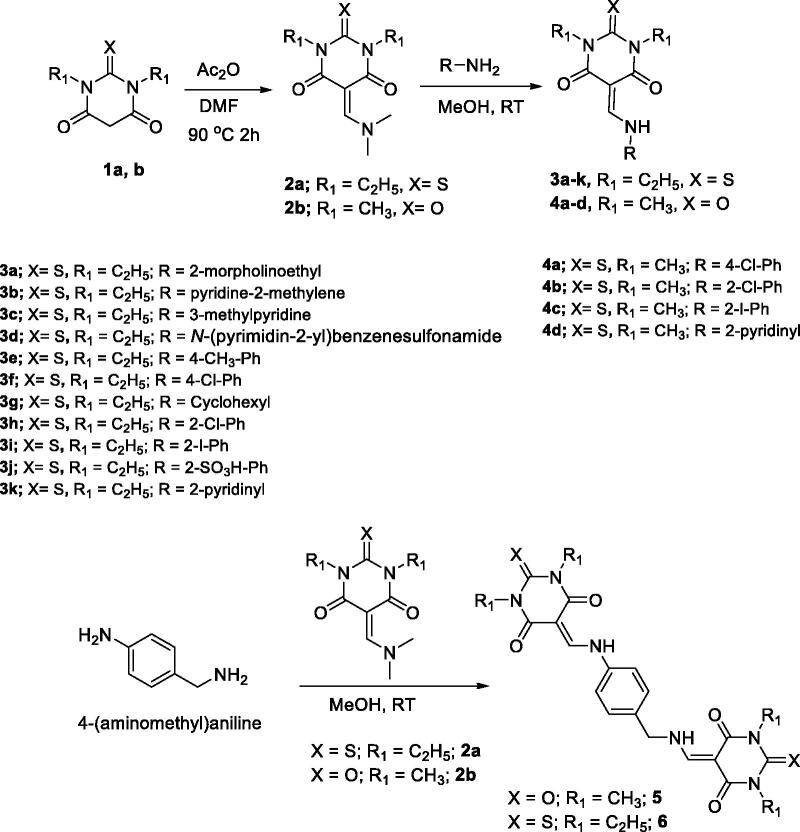
Synthetic route for the synthesis of **3a–k**, **4a–d**, **5**, and **6**.

Enamine derivatives **2a**^32^ and **2b**^33^ were prepared by reacting the commercially available compounds, 1,3-diethylthiobarbituric acid **1a** or 1,3-dimethylbarbituric acid **1b** with DMF in the presence of acetic anhydride as solvent for 2 h at 90 °C to afford **2a** and **2b**, respectively as a yellow crystalline solid in good yields. Compounds **2a**^32^ and **2b**^33^ were reacted with different amines in ethanol at RT to afford the target products **3a–k** and **4a–d**, respectively (Scheme 1) in excellent yields and purities, as observed from their spectral data. The reaction of **2a** (2 equiv.) or its analogues **2b** (2 equiv.) with the commercially available material 4-(aminomethyl)aniline (1 equiv.) under the same conditions described above gave the dimeric products **6** and **5**, respectively as shown in Scheme 1. The structures of the products obtained were deduced by ^1^H- and 13C-NMR spectra (Supplementary material).

### 2.2. Biological activity

All the synthesised derivatives of TBA (**3a**–**k)**, BA (**4a–d)**, and the dimeric analogues **5** and **6** were evaluated for their capacity to inhibit α-glucosidase and protein glycation *in vitro* in comparison to acarbose (IC_50_ = 875.75 ± 2.08 µM) and rutin (IC_50_ = 54.59 ± 2.20 µM), as standard tested compounds (Table 1).

**Table 1.** Result of *in vitro* α-glucosidase enzyme inhibitor and anti-glycation activities.

Compound	Structure	Anti-glycation assay IC_50_ ± SEM (µM)	α-Glucosidase IC_50_ ± SEM (µM)
**3a**		88.57 ± 0.37^b^	NA
**3b**		80.36 ± 0.74^b^	NA
**3c**		82.22 ± 4.36^b^	NA
**3d**		130.53 ± 3.15^b^	NA
**3e**		77.28 ± 0.72^b^	NA
**3f**		81.74 ± 1.39^b^	NA
**3g**		82.36 ± 5.09^b^	397.45 ± 0.98^a^
**3h**		70.92 ± 1.84^b^	NA
**3i**		75.13 ± 0.65^b^	264.07 ± 1.87^a^
**3j**		101.92 ± 1.7^b^	433.33 ± 2.34^a^
**3k**		ND	ND
**4a**		NA	NA
**4b**		NA	NA
**4c**		NA	NA
**4d**		NA	NA
**5**		554.76 ± 9.1^b^	448.63 ± 2.46^a^
**6**		31.5 ± 0.81^a^	NA
**Rutin**		**54.59** ± **2.20**	–
**Acarbose**		–	**875.75** ± **2.08**

^a^ Significant activity.

^b^ Moderate activity.

ND: not determined; NA: not active.

The results summarised in Table 1 indicated that all the *N,N′*-dimethylbarbituric-based enamine acid derivatives **4a–d** were completely inactive, while compounds **3a–k**, **5**, and **6** showed moderate to significant activity against protein glycation (IC_50_ = 554.76 ± 9.1 to 31.5 ± 0.81 µM). The dimeric moiety of TBA **6** *via* diaminobenezene linkage (IC_50_ = 31.5 ± 0.81 µM, Table 1) was the most protein glycation inhibitor in this series of compounds, and showed more activity than the standard rutin (IC_50_ = 54.59 ± 2.20 µM). While, the dimeric analogues of BA **5** (IC_50_ = 554.76 ± 9.1 µM) was the least active.

On the other hand, substituted phenyl with an electron-withdrawing group such as a chlorine atom (a weak deactivating group) at the *ortho* position, showed a better anti-glycation activity than the same atom at the *para* position. Therefore, the change in the position had a remarkable effect on the anti-glycation activity^34^ (**3 h** *vs* **3f**, Table 1). Halogen with a higher atomic weight and weaker electron-withdrawing effect, such as iodine at the *ortho* position, decreased the activity as compared to chlorine at the same position (**3i** *vs* **3 h**). This observation could be attributed to the negative inductive effect^35^^,^^36^. In contrast, a strong electron-withdrawing group, such as sulphonic acid at *ortho* position, decreased the activity compared to chlorine and iodine in the *ortho* position (**3j** *vs* **3 h**). Electron-donating group such as methyl (a weak donating group) at the *para* position yielded slightly better and a moderate activity as compared to the chlorine at the same position (**3e** *vs* **3f**). On the other hand, replacing the 4-methylphenyl **3e** by 2-pyridylmethylene **3b** or 3-methylpyridyl **3c** decreased the anti-glycation activity, and showed a comparable activity to compounds **3g** and **3f** as shown in Table 1. Compound with pyrimidine benzenesulfonamide **3d** moiety decreased the activity, which is consistent with the result obtained for **3j** with a strong withdrawing group. While, compounds with 2-morpholinoethyl **3a** and cyclohexyl **3g** moieties showed moderate activity against protein glycation.

The results summarised in Table 1 indicated, once again, that none of the BA enamine derivatives showed any activity, while **3g**, **3i**, **3j**, and **5** exerted a significant activity against α-glucosidase (IC_50_ = 264.07 ± 1.87 to 448.63 ± 2.46 µM). Of the series of compounds, thiopyrimidine trione derivative with higher atomic weight halogen, such as iodine at the *ortho* position, was the most active, exhibiting 3.3-fold higher activity than the standard acarbose. Compounds with a cyclohexyl ring **3g**, sulphonic acid **3j**, and the dimeric analogue of BA **5** showed twice the activity of the standard drug. The rest of the compounds did not show any activity.

**Figure 1.**
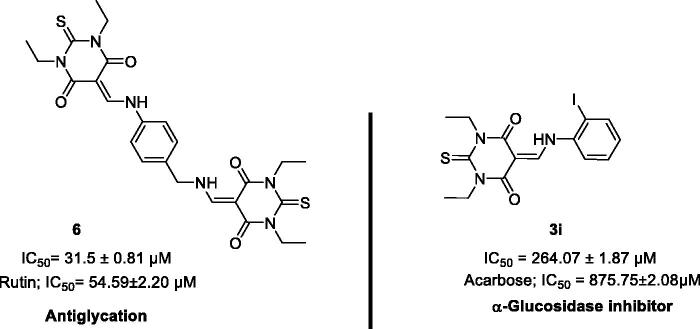
Lead compounds **3i** and **6** with promising activities.

Finally, the most two active compounds from the series are shown in Figure 1. In conclusion, this work has demonstrated that the core of TBA-based enamine derivatives is a privileged structure for anti-glycation and α-glucosidase inhibition and thus deserves further investigation.

### 2.3. Molecular docking studies

Molecular docking provides significant insight into ligand-protein binding modes and mechanisms. Here, molecular docking studies were carried out to explore the binding modes of TBA derivatives with a notorious α-glucosidase, such as that of Baker’s yeast (*Saccharomyces cerevisiae*). We used our previously built homology model of α-glucosidase from the template (PDB ID: 3A4A)^30^. Initially, the 3 D structures of all the ligands were built, protonated, and minimised by means of the MMFF94x force field^37^, and using the molecular operating environment (MOE)^38^ 2018.04. All recently synthesised TBA derivatives and a reference inhibitor (acarbose) were docked into the active site of the receptor using the default parameters in MOE. Each complex was visually analysed for ligand–protein interactions, and their images were prepared using UCSF chimaera software^39^.

**Figure 2.**
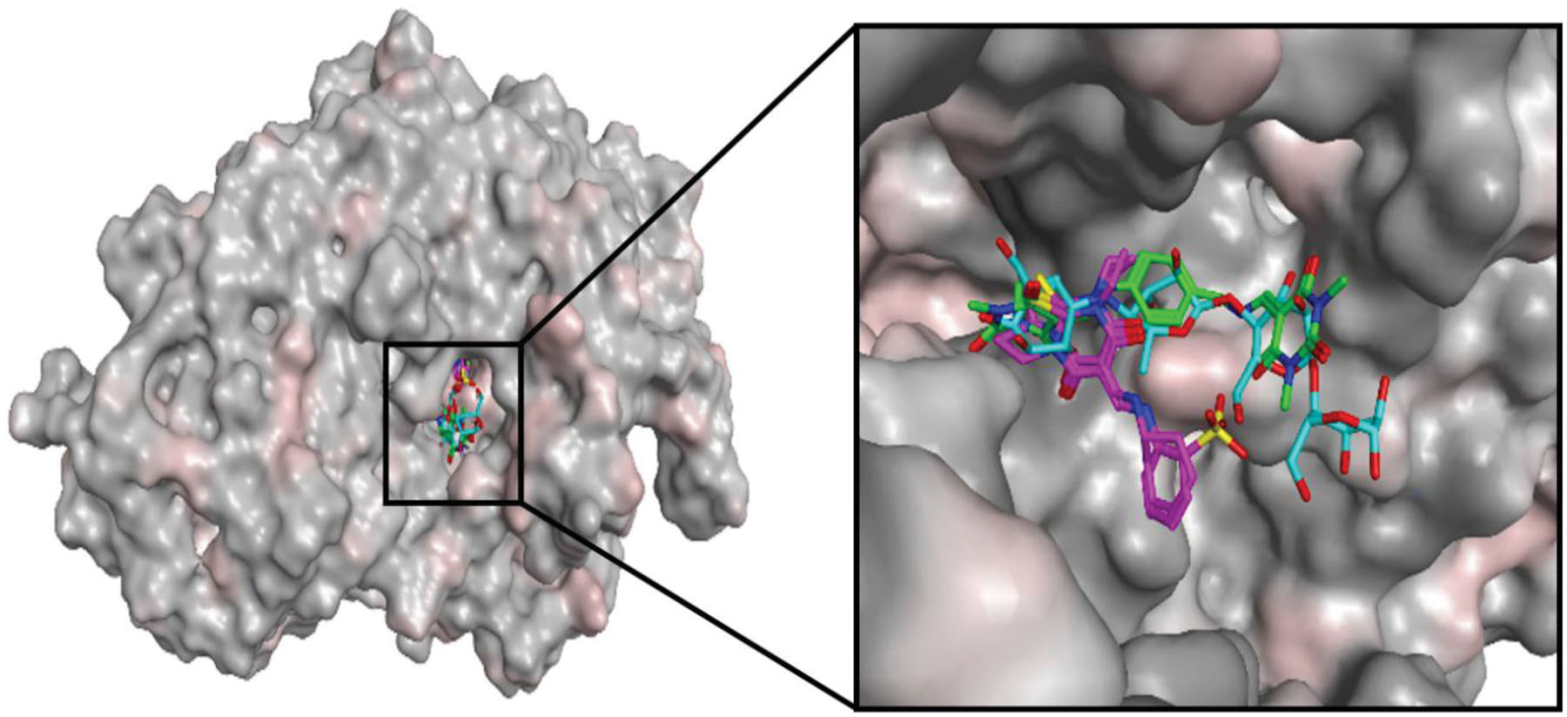
Binding mode of thiobarbituric acid derivatives into the α-glucosidase binding cavity. For clarity, acarbose is shown in cyan. Compounds **3g, 3i**, and **3j** are indicated in pink, and **5** in green. The part of the enzyme in the background is shown as surface model.

The top ranked conformer of TBA derivatives and standard (acarbose) were selected based on docking score. The docking score of the ligands **3g**, **3i**, **3j**, and **5** and acarbose were −3.081, −4.909, −5.19, −5.642, and −4.382, respectively. The docking study revealed that the acarbose, and all the ligands accommodated into the binding pocket of the C-terminal domain of α-glucosidase. The clustering of standard and synthetic compounds at the allosteric site of the C-terminal domain is shown in Figure 2.

**Figure 3.**
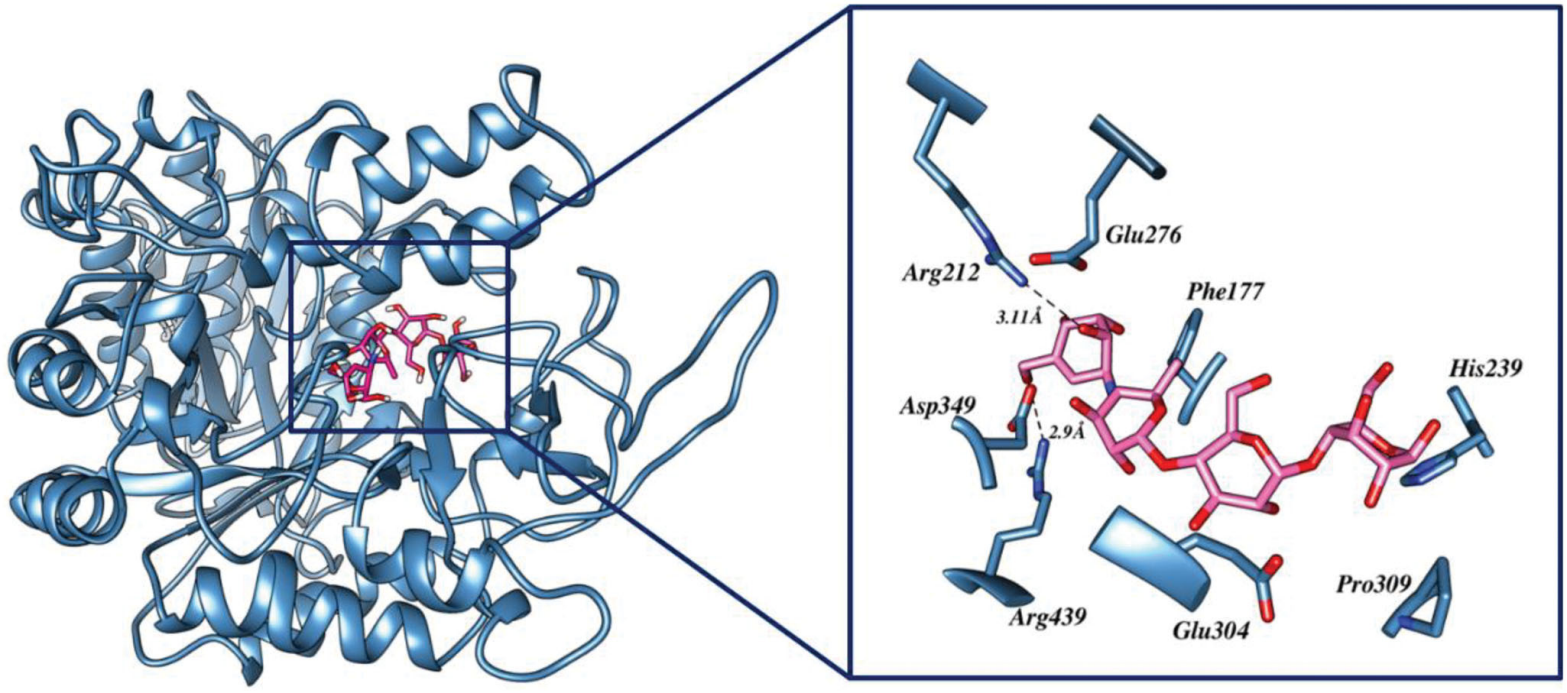
Interactions of acarbose with crucial residues of α-glucosidase.

Acarbose occupied a large cavity in the binding sites of α-glucosidase due to its larger size, as compared to the synthetic compounds. The oxygen functionality of acarbose formed two hydrogen bonds with the active site residues, Arg212 and Arg439. Ring structures were involved in the π–π interactions with Phe177, His239, and Pro309. Moreover, residues Glu276, Glu304, and Asp349 interacted hydrophobically with the ligand (Figure 3).

**Figure 4.**
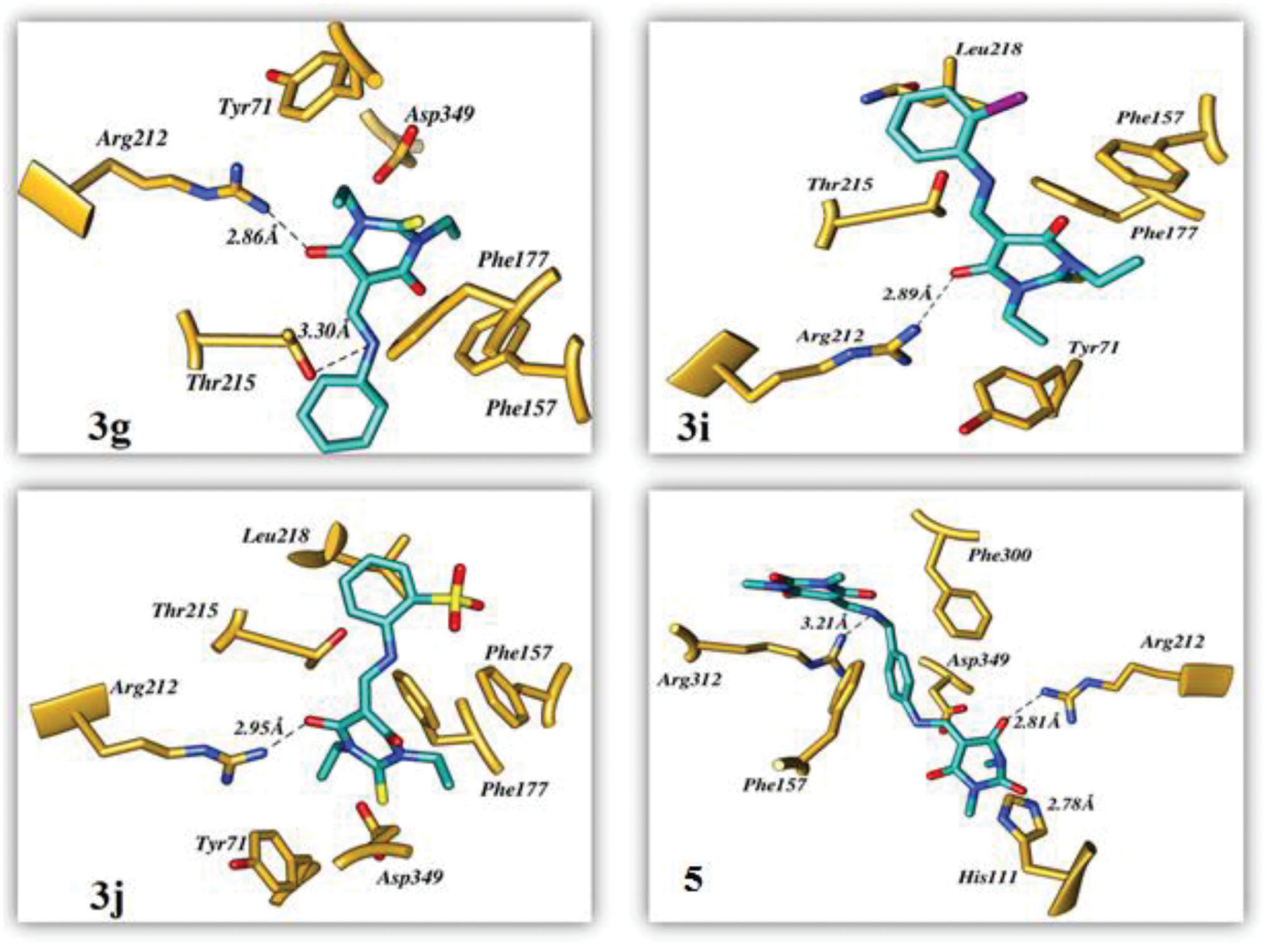
The predicted binding interactions of compounds **3g**, **3i**, **3j**, and **5** in the active site.

The carbonyl oxygen of the thiobarbituric ring of **3g, 3i,** and **3j** showed hydrogen bond interactions with crucial residue Arg212. Another hydrogen bond was observed between the nitrogen atoms of **3g** with Thr215. These compounds were further stabilised through π–π interactions with the crucial active site residues Tyr71, Phe157, and Phe177. Additionally, π–π interactions were observed with the thiol ring of **3j** through Phe177 and Tyr71. In the case of compound **3i,** Phe157 was involved in forming halogen–π interactions. Moreover, hydrophobic interactions with the active site residues Phe157, Thr215, Leu218, and Arg349 stabilised these compounds. In the case of **5**, the 2,4,6-trione ring-bearing oxygen atom formed hydrogen bonds with His111 and Arg212. Meanwhile, the amine functionality of the ligand also formed a hydrogen bond with residue Arg312. The benzene ring was involved in π–π interactions with Phe157 and Phe300. The hydrophobic interaction with crucial residue Arg349 also contributed to the binding of **5** with α-glucosidase. The interaction diagrams of all the ligands are shown in Figure 4. The docking results of **5** were in good agreement with experimental results, thereby indicating that it could be a good candidate as α-glucosidase inhibitor.

## 3. Conclusions

Several derivatives of barbitutic and thiobarbituric enamine derivatives were synthesised, characterised, and screened for *in vitro* evaluation of α-glucosidase enzyme inhibition and anti-glycation activity. The results reveal that the four monomeric compounds **4a–d** derived from *N,N′*-dimethylbarbituric enamine derivatives showed no anti-glycation activity, while compounds derived from *N,N′*-diethylthiobarbituric enamine derivatives **3a–k** exhibited moderate activity against protein glycation with IC_50_ in the range 70–550 µM. The most potent anti-glycation activity was showed by the dimeric product from *N,N′*-diethylthiobarbituric enamine **6** with an IC_50_ of 31.5 µM, while the dimeric analogue of *N,N′*-dimethylbarbituric enamine **5** showed less activity with an IC_50_ of 554.8 µM. The reported series of compounds were found to inhibit α-glucosidase activity in a reversible mixed-type manner with IC_50_ between 264 and 448 µM. The type and position of substituent on phenyl ring (enamine moiety) has great impact on the biological activity. In this regard, the moderate electron-withdrawing group, such as a chlorine atom at the *ortho* position **3 h** showed greater activity compared to the same atom at the *para* position **3f**. On the other hand, the presence of iodine at the *ortho* position decreased activity compared to chlorine in the same position (**3i** *vs* **3 h**). The strong electron-withdrawing group, such as sulphonic acid showed decrease in activity compared to weak electron-donating group like methyl (**3e**).

The dimeric thiobarbituric derivative **6** showed better anti-glycation activity compared with the standard rutin, while thiobarbituric *ortho*-iodo-enamine derivative **3i** showed a positive effect as α-glucosidase inhibitor compared to the standard acarbose.

Molecular docking studies indicated that compounds of **3g**, **3i**, **3j**, and **5** are located close to the active site of α-glucosidase, which may cover the active pocket, thereby inhibiting the binding of the substrate to the enzyme.

This work has confirmed that the core of (thio)barbituric-based enamine derivatives are a privileged structure, because in addition of the previous described biological activity, they have shown activity for anti-glycation and α-glucosidase inhibition.

## 4. Experimental

### 4.1. General methods

All melting points were determined using Mel-Temp apparatus and are uncorrected. Thin layer chromatography (TLC) was performed on silica gel (Kiesel gel G, Merck) and spots were detected under UV light at 254 nm. FTIR Spectra were recorded in a KBr matrix on a Bruker Tensor 37 FTIR spectrophotometer. ^1^H-NMR spectra were recorded with a JEOL 400 MHz, 13C-NMR were recorded using the JEOL spectrophotometers, and the chemical shifts (*δ*) are given in ppm.

### 4.2. General procedure for the synthesis of 3a–k, 4a–d, 5, and 6

A solution of **2a**^32^ or **2b**^33^ (1 equiv.) was mixed with different amines (1 equiv.) in MeOH (10 ml) and stirred at room temperature for 10–120 min (TLC 20% EtOAc/n-hexane). The solvent was evaporated slowly, providing the corresponding solid products in excellent yields and purities.

#### 4.2.1. 1,3-Diethyl-5-(((2-morpholinoethyl)amino)methylene)-2-thioxodihydropyrimidine-4,6(1H,5H)-dione (3a)

Compound **3a** was synthesised from **2a** and 4–(2-aminoethyl)morpholine following the general procedure, affording the product as a yellow powder in 81% yield; mp 135 °C; IR (KBr, cm^−1^): 3420, 2999, 2960, 2908, 2870, 1624, 1591, 1456; ^1^H-NMR (CDCl_3_, *δ,* ppm): 10.60 (brs, 1H, NH), 8.23 (d, 1H, *J* = 14.8 Hz, CH=), 4.50 (m, 4H, 2CH_2_), 3.72 (q, 2H, CH_2_), 3.54 (m, 4H, 2CH_2_), 2.60 (m, 2H, CH_2_), 2.50 (m, 4H, 2CH_2_), 1.25 (m, 6H, 2CH_3_); ^13^C-NMR (CDCl_3_ *δ,* ppm): 179.1, 163.0, 161.2, 160.6, 93.0, 66.9, 57.6, 53.6, 47.1, 43.0, 42.3, 12.5, 12.4; LC/MS (ESI): 341.44 [M + 1]^+^; Anal. Calcd for C_15_H_24_N_4_O_3_S: C, 52.92; H, 7.11; N, 16.46; Found: C, 53.01; H, 7.25; N, 16.59.

#### 4.2.2. 1,3-Diethyl-5-(((pyridin-2-ylmethyl)amino)methylene)-2-thioxodihydropyrimidine-4,6(1H,5H)-dione (3b)

Compound **3b** was synthesised from **2a** and 2-picolylamine following the general procedure, affording the product as a pink powder in 83% yield; mp 154 °C; IR (KBr, cm^−1^): 3215, 3045, 2958, 2908, 2866, 1614, 1598, 1544,1463; ^1^H-NMR (CDCl_3_ *δ,* ppm): 11.01 (brs, 1H, NH), 8.63 (d, 1H, *J* = 5.2 Hz, Ar-H) , 8.39 (d, 1H, *J* = 14.0 Hz, CH=), 7.72 (t, 1H, *J* = 8.8 Hz, Ar-H), 7.27 (d, 1H, *J* = 8.8 Hz, Ar-H), 7.23 (m, 1H, Ar-H), 4.76 (d, 2H, *J* = 3.6 Hz, CH_2_), 4.56 (m, 4H, 2CH_2_), 1.28–124 (m, 6H, *J* = 16.4 Hz, 2CH_3_); ^13^C-NMR (CDCl_3_ *δ,* ppm): 179.1, 163.0, 161.2, 160.7, 154.1, 150.3, 137.3, 123.5, 121.7, 93.5, 55.1, 43.0, 42.3, 12.5, 12.4; LC/MS (ESI): 319.40 [M + 1]^+^; Anal. Calcd for C_15_H_18_N_4_O_2_S: C, 56.59; H, 5.70; N, 17.60; Found: C, 56.72; H, 5.81; N, 17.78.

#### 4.2.3. 1,3-Diethyl-5-(((4-methylpyridin-2-yl)amino)methylene)-2-thioxodihydro pyrimidine-4,6(1H,5H)-dione (3c)

Compound **3c** was synthesised from **2a** and 2-amino-4-picoline following the general procedure, affording the product as a light yellow powder in 87% yield; mp 175 °C; IR (KBr, cm^−1^) 3215, 3157, 3045, 2958, 2908, 2866, 1614, 1598, 1544, 1463; ^1^H-NMR (CDCl_3_ *δ,* ppm):12.25 (d, 1H, *J* = 13.2 Hz, NH), 9.40 (d, 1H, *J* = 13.2 Hz, CH=), 8.27 (d, 1H, *J* = 5.2 Hz, Ar-H), 6.98 (d, 1H, *J* = 5.2 Hz, Ar-H), 6.86(s, 1H, Ar-H), 4.55 (m, 4H, 2CH_2_), 2.38 (s, 3H, CH_3_), 1.29 (m, 6H, 2CH_3_); ^13^C-NMR (CDCl_3_ *δ,* ppm):179.1, 163.3, 160.7, 152.5, 150.7, 149.6, 149.0, 122.9, 113.6, 95.8, 43.2, 42.5, 21.2, 12.5, 12.4; LC/MS (ESI): 319.40 [M + 1]^+^; Anal. Calcd for C_15_H_18_N_4_O_2_S: C, 56.59; H, 5.70; N, 17.60; Found: C, 56.81; H, 5.78; N, 17.79.

#### 4.2.4. 4-(((1,3-Diethyl-4,6-dioxo-2-thioxotetrahydropyrimidin-5(2H)-ylidene)methyl)amino)-N-(pyrimidin-2-yl)benzenesulfonamide (3d)

Compound **3d** was synthesised from **2a** and sulphadiazine following the general procedure, affording the product as a yellow powder in 85% yield; mp 204 °C; IR (KBr, cm^−1^): 3421, 3116, 2958, 2860, 1618, 1591, 1508, 1440; ^1^H-NMR (DMSO-d_6_, *δ*, ppm): 12.20 (d, 1H, *J* = 14.0 Hz, NH), 8.72 (d, 1H, *J* = 14.0 Hz, NH), 8.52 (d, 1H, *J* = 4.4 Hz, CH=), 8.47 (d, 1H, *J* = 8.8 Hz, Ar-H), 8.0 (d, 1H, *J* = 8.8 Hz, Ar-H), 7.79 (d, 2H, *J* = 8.8 Hz, Ar-H), 7.10 (m, 1H, Ar-H), 6.57 (d, 2H, *J* = 8.8 Hz, Ar-H), 4.42 (m, 4H, 2CH_2_), 1.21 (m, 6H, 2CH_3_); ^13^C-NMR (DMSO-d_6_, *δ*, ppm): 178.9, 162.2, 160.5, 158.8, 157.8, 157.3, 154.3, 153.6, 142.4, 130.4, 129.8, 125.4,119.8, 116.1, 112.7, 95.7, 42.9, 42.4, 12.8, 12.7; LC/MS (ESI): 461.53 [M + 1]^+^; Anal. Calcd for C_19_H_20_N_6_O_4_S_2_: C, 49.55; H, 4.38; N, 18.25; Found: C, 49.66; H, 4.50; N, 18.41.

#### 4.2.5. 1,3-Diethyl-2-thioxo-5-((p-tolylamino)methylene)dihydropyrimidine-4,6(1H,5H)-dione (3e)

Compound **3e** was synthesised from **2a** and 4-methylanline uracil following the general procedure, affording the product as a yellow powder in 89% yield; mp 139 °C; IR (KBr, cm^−1^): 3448, 3215, 3169, 2953, 2866, 1595, 1570, 1554, 1476, 1435, 1440; ^1^H-NMR (CDCl_3_, *δ*, ppm): 12.32 (d, 1H, *J* = 13.8 Hz, NH), 8.70 (d, 1H, *J* = 14.0 Hz, CH=), 7.27 (d, 2H, *J* = 8.0 Hz, Ar-H), 7.22 (dd, 2H, *J* = 8.0 Hz, Ar-H), 4.60(m, 4H, 2CH_2_), 2.36 (s, 3H, CH_3_), 1.30 (m, 6H, 2CH_3_); ^13^C-NMR (CDCl_3_, *δ*, ppm): 178.8, 163.2, 160.9, 152.9, 137.3, 135.5, 130.9, 118.2, 94.6, 43.2, 42.5, 12.4, 12.2; LC/MS (ESI): 317.41 [M + 1]^+^; Anal. Calcd for C_16_H_19_N_3_O_2_S: C, 60.55; H, 6.03; N, 13.24; Found: C, 60.32; H, 6.00; N, 13.43.

#### 4.2.6. 5-(((4-Chlorophenyl)amino)methylene)-1,3-diethyl-2-thioxodihydropyrimidine-4,6(1H,5H)-dione (3f)

Compound **3f** was synthesised from **2a** and 4-chloroanline following the general procedure, affording the product as a yellow powder in 78% yield; mp 215 °C; IR (KBr, cm^−1^): 3302, 2958, 2908, 2866, 1614, 1587, 1545, 1504, 1438, 1409; ^1^H-NMR (CDCl_3_, *δ*, ppm): 12.32 (d, 1H, *J* = 14.0 Hz, NH), 8.65 (d, 1H, *J* = 14.0 Hz, CH=), 7.38 (d, 2H, *J* = 8.0 Hz, Ar-H), 7.25 (d, 2H, *J* = 8.8 Hz, Ar-H), 4.55 (m, 4H, 2CH_2_), 1.30 (t, 6H, *J* = 7.9 Hz, 2CH_3_); ^13^C-NMR (CDCl_3,_ *δ*, ppm): 178.9, 163.2, 160.8, 152.8, 136.6, 132.6, 130.4, 119.4, 95.3, 43.2, 42.5, 12.5, 12.3; LC/MS (ESI): 338.82 [M + 1]^+^; Anal. Calcd for C_15_H_16_ClN_3_O_2_S: C, 53.33; H, 4.77; N, 12.44; Found: C, 53.54; H, 4.80; N, 12.63.

#### 4.2.7. 5-((Cyclohexylamino)methylene)-1,3-diethyl-2-thioxodihydropyrimidine-4,6(1H,5H)-dione (3g)

Compound **3g** was synthesised from **2a** and cyclohexylamine following the general procedure, affording the product as a white powder in 84% yield; mp 105 °C; IR (KBr, cm^−1^): 3302, 2958, 2908, 2866, 1614, 1587, 1545, 1504, 1438, 1409; ^1^H-NMR (DMSO-d_6_, *δ*, ppm): 10.61 (brs, 1H, NH), 8.25 (d, 1H, *J* = 15.6 Hz, CH=), 4.52 (m, 4H, 2CH_2_), 3.40 (m, 1H, CH), 1.98 (m, 2H, CH_2_), 1.83 (m, 2H, CH_2_), 1.78 (m, 2H, CH_2_), 1.47 (m, 2H, 2CH_2_), 1.28 (m, 2H, 2CH_3_); ^13^C-NMR (DMSO-d_6_, *δ*, ppm): 179.0, 163.2, 161.3, 158.4, 92.6, 59.3, 42.9, 42.3, 33.5, 24.9, 24.3, 12.5, 12.4; LC/MS (ESI): 310.43 [M + 1]^+^; Anal. Calcd for C_15_H_23_N_3_O_2_S: C, 58.23; H, 7.49; N, 13.58; Found: C, 58.39; H, 7.53; N, 13.78.

#### 4.2.8. 5-(((2-Chlorophenyl)amino)methylene)-1,3-diethyl-2-thioxodihydropyrimidine-4,6(1H,5H)-dione (3 h)

Compound **3h** was synthesised from **2a** and 2-chloroanline following the general procedure, affording the product as a yellow powder in 89% yield; mp 160 °C; IR (KBr, cm^−1^): 3302, 2958, 2908, 2866, 1614, 1587, 1545, 1504, 1438, 1409; ^1^H-NMR (CDCl_3_, *δ*, ppm): 12.62 (d, 1H, *J* = 13.2 Hz, NH), 8.73 (d, 1H, *J* = 14.0 Hz, CH=), 7.49 (m, 2H, Ar-H), 7.39 (t, 1H, *J* = 7.9 Hz, Ar-H), 7.21 (t, 1H, *J* = 8.0 Hz, Ar-H), 4.56 (m, 4H, 2CH_2_), 1.30(m, 6H, 2CH_3_); ^13^C-NMR (CDCl_3_, *δ*, ppm): 178.9, 163.0, 160.9, 152.1, 135.3, 130.7, 128.5, 127.3, 125.0, 116.9, 96.1, 43.2, 42.6, 12.5, 12.4; LC/MS (ESI): 338.82 [M + 1]^+^; Anal. Calcd for C_15_H_16_ClN_3_O_2_S: C, 53.33; H, 4.77; N, 12.44; Found: C, 53.53; H, 4.92; N, 12.60.

#### 4.2.9. 1,3-Diethyl-5-(((2-iodophenyl)amino)methylene)-2-thioxodihydropyrimidine-4,6(1H,5H)-dione (3i)

Compound **3i** was synthesised from **2a** and 2-iodoanline following the general procedure, affording the product as a yellow powder in 83% yield; mp 175 °C; IR (KBr, cm^−1^): 3302, 2958, 2908, 2866, 1614, 1587, 1545, 1504, 1438, 1409; ^1^H-NMR (CDCl_3_, *δ*, ppm): 12.42 (d, 1H, *J* = 13.2 Hz, NH), 8.67 (d, 1H, *J* = 14.0 Hz, CH=), 791 (d, 1H, *J* = 7.2 Hz, Ar-H), 7.45 (t, 1H, *J* = 7.9 Hz, Ar-H), 7.36 (d, 1H, *J* = 8.0 Hz, Ar-H), 6.98 (t, 1H, *J* = 7.2 Hz, Ar-H), 4.53 (m, 4H, 2CH_2_), 1.30 (m, 6H, 2CH_3_); ^13^C-NMR (CDCl_3_, *δ*, ppm): 179.0, 162.8, 160.9, 153.0, 139.9, 130.0, 128.2,125.1, 117.7, 95.9, 89.9, 43.2, 42.5, 12.6, 12.4; LC/MS (ESI): 430.28 [M + 1]^+^; Anal. Calcd for C_15_H_16_IN_3_O_2_S: C, 41.97; H, 3.76; N, 9.79; Found: C, 41.88; H, 3.81; N, 10.01.

#### 4.2.10. 2-(((1,3-Diethyl-4,6-dioxo-2-thioxotetrahydropyrimidin-5(2H)-ylidene)methyl) amino)benzenesulfonic acid (3j)

Compound **3j** was synthesised from **2a** and 2-aminobenzenesulfonic acid following the general procedure, affording the product as a yellow powder in 80% yield; mp 243 °C; IR (KBr, cm^−1^): 3302, 2958, 2908, 2866, 1614, 1587, 1545, 1504, 1438, 1409; ^1^H-NMR (DMSO-d_6_, *δ*, ppm): 13.01 (d, 1H, *J* = 14.8 Hz, NH), 8.62 (d, 1H, *J* = 14.4 Hz, CH=), 7.78 (d, 1H, *J* = 7.9 Hz, Ar-H), 7.64 (d, 1H, *J* = 8.0 Hz, Ar-H), 7.49 (t, 1H, *J* = 8.6 Hz, Ar-H), 7.30 (t, 1H, *J* = 8.4 Hz, Ar-H), 4.43 (m, 4H, 2CH_2_), 1.20 (m, 6H, 2CH_3_); ^13^C-NMR (DMSO-d_6_, *δ*, ppm): 178.9, 161.1, 160.9, 153.9, 138.6, 135.9, 130.7, 128.1, 124.6, 118.3, 95.3, 42.9, 42.2, 12.8. LC/MS (ESI): 384.44 [M + 1]^+^; Anal. Calcd for C_15_H_17_N_3_O_5_S_2_: C, 46.99; H, 4.47; N, 10.96; Found: C, 47.09; H, 4.53; N, 11.13.

#### 4.2.11. 1,3-diethyl-5-((pyridin-2-ylamino)methylene)-2-thioxodihydropyrimidine-4,6(1H,5H)-dione (3k)

Compound **3k** was synthesised from **2a** and pyridin-2-amine following the general procedure, affording the product as a yellow powder in 83% yield; mp 243 °C; IR (KBr, cm^−1^): 3302, 2958, 2908, 2866, 1614, 1587, 1545, 1504, 1438, 1409; ^1^H-NMR (DMSO-d_6_, *δ*, ppm): 13.03 (d, 1H, *J* = 14.8 Hz, NH), 8.62 (d, 1H, *J* = 14.4 Hz, CH=), 7.76 (d, 1H, *J* = 7.6 Hz, Ar-H), 7.63 (d, 1H, *J* = 8.8 Hz, Ar-H), 7.49 (t, 1H, *J* = 9.6 Hz, Ar-H), 7.28 (t, 1H, *J* = 8.4 Hz, Ar-H), 4.46 (m, 4H, 2CH_2_), 1.22 (m, 6H, 2CH_3_); ^13^C-NMR (DMSO-d_6_, *δ*, ppm): 178.4, 160.5, 159.2, 138.1, 135.6, 130.7, 118.3, 94.6, 42.4, 41.7, 12.2. LC/MS (ESI): 305.35 [M + 1]^+^; Anal. Calcd for C_14_H_16_N_4_O_2_S: C, 55.25; H, 5.30; N, 18.41; Found: C, 55.38; H, 5.41; N, 18.59.

#### 4.2.12. 5-(((4-Chlorophenyl)amino)methylene)-1,3-dimethylpyrimidine-2,4,6(1H,3H,5H)-trione (4a)

Compound **4a** was synthesised from **2b** and 4-chloroanline following the general procedure, affording the product as a white powder in 87% yield; mp 197 °C; IR (KBr, cm^−1^): 3302, 2958, 2908, 2866, 1614, 1587, 1545, 1504, 1438, 1409;^1^H-NMR (CDCl_3_, *δ*, ppm):12.00 (d, 1H, *J* = 13.6 Hz, NH), 8.60 (d, 1H, *J* = 14.0 Hz, CH=), 7.36 (d, 2H, *J* = 8.8 Hz, Ar-H), 7.16 (d, 2H, *J* = 8.8 Hz, Ar-H), 3.32 (s, 6H, 2CH_3_); ^13^C-NMR (CDCl_3_, *δ*, ppm): 165.1, 162.6, 151.8, 136.8, 132.1, 130.3,119.2, 93.4, 28.1, 27.4; LC/MS (ESI): 294.71 [M + 1]^+^; Anal. for C_13_H_12_ClN_3_O_3_; Calcd: C, 53.16; H, 4.12; N, 14.31; Found: C, 53.15; H, 4.12; N, 14.33.

#### 4.2.13. 5-(((2-Chlorophenyl)amino)methylene)-1,3-dimethylpyrimidine-2,4,6(1H,3H,5H)-trione (4b)

Compound **4b** was synthesised from **2b** and 2-chloroanline following the general procedure, affording the product as a white powder in 87% yield; mp 202 °C; IR (KBr, cm^−1^): 3637, 3423, 3197, 2960, 2935, 1583, 1570, 1510, 1462; ^1^H-NMR (CDCl_3_, *δ*, ppm): 12.44 (d, 1H, *J* = 12.8 Hz, NH), 8.73 (d, 1H, *J* = 13.2 Hz, CH=), 7.48 (m, 2H, Ar-H), 7.36 (t, 1H, *J* = 7.2 Hz, Ar-H), 7.18 (m, 1H, Ar-H), 3.38 (s, 3H, CH_3_), 3.36 (s, 3H, CH_3_); ^13^C-NMR (CDCl_3_, *δ*, ppm): 164.9, 162.7, 151.9, 151.1, 135.4, 130.6, 128.5, 126.9, 124.7, 116.6, 94.4, 28.2, 27.5; LC/MS (ESI): 294.71 [M + 1]^+^; Anal. for C_13_H_12_ClN_3_O_3_; Calcd: C, 53.16; H, 4.12; N, 14.31; Found: C, 53.17; H, 4.11; N, 14.29.

#### 4.2.14. 5-(((2-Iodophenyl)amino)methylene)-1,3-dimethylpyrimidine-2,4,6(1H,3H,5H)-trione (4c)

Compound **4c** was synthesised from **2b** and 2-iodoanline following the general procedure, affording the product as a white powder in 89% yield; mp 285 °C; IR (KBr, cm^−1^): 3086, 3053, 2953, 2885,1598, 1560, 1516, 1463, 1371; ^1^H-NMR (CDCl_3_, *δ*, ppm): 12.77 (d, 1H, *J* = 14.8 Hz, NH), 8.55 (d, 1H, *J* = 14.0 Hz, CH=), 7.77 (d, 1H, *J* = 7.2 Hz, Ar-H), 7.57 (d, 1H, *J* = 8.0 Hz, Ar-H), 7.46 (t, 1H, *J* = 7.6 Hz, Ar-H), 7.26 (t, 1H, *J* = 7.2 Hz, Ar-H), 3.20 (s, 6H, 2CH_3_); ^13^C-NMR (CDCl_3_, *δ*, ppm):163.3, 162.9, 152.2, 151.9, 138.3, 136.4, 131.3, 128.1, 125.9, 118.5, 93.9, 28.2, 27.6; LC/MS (ESI): 386.16 [M + 1]^+^; Anal. for C_13_H_12_IN_3_O_3_; Calcd: C, 40.54; H, 3.14; N, 10.91; Found: C, 40.55; H, 3.15; N, 10.90.

#### 4.2.15. 1,3-Dimethyl-5-((pyridin-2-ylamino)methylene)pyrimidine-2,4,6(1H,3H,5H)-trione (4d)

Compound **4d** was synthesised from **2b** and 2-aminopyridine following the general procedure, affording the product as a white powder in 85% yield; mp 287–290 °C; IR (KBr, cm^−1^): 3420, 2999, 2960, 2908, 2870, 1624, 1591, 1456; ^1^H-NMR (CDCl_3_, *δ*, ppm): 12.11 (brs, 1H, NH), 9.41 (d, 1H, *J* = 13.2 Hz, CH=), 8.43 (d, 1H, *J* = 4.4 Hz, Ar-H), 7.75 (dd, 1H, *J* = 8.0, 2.4 Hz, Ar-H), 7.16 (t, 1H, *J* = 7.6, Hz, Ar-H), 6.99 (d, 2H, *J* = 8.0 Hz, Ar-H), 3.36 (s, 6H, 2CH_3_); ^13^C-NMR (CDCl_3_, *δ*, ppm): 165.3, 162.6, 152.0, 151.3, 149.7, 149.3, 138.9, 121.4, 112.7, 94.3, 28.2, 27.5; LC/MS (ESI): 261.25 [M + 1]^+^; Anal. for C_12_H_12_N_4_O_3_; Calcd: C, 55.38; H, 4.65; N, 21.53; Found: C, 55.38; H, 4.64; N, 21.51.

#### 4.2.16. 5-(((4-(((1,3-Dimethyl-2,4,6-trioxotetrahydropyrimidin-5(2H)-ylidene)methyl) amino)benzyl)amino)methylene)-1,3-dimethylpyrimidine-2,4,6(1H,3H,5H)-trione (5)

Compound **5** was synthesised from **2b** (2 equiv.) and 4-aminobenzylamine (1 equiv.) following the general procedure, affording the product as a white powder in 90% yield; mp 195 °C; IR (KBr, cm^−1^): 3302, 2958, 2908, 2866, 1614, 1587, 1545, 1504, 1438, 1409; ^1^H-NMR (DMSO-d_6_, *δ*, ppm): 10.48 (brs, 2H, NH), 8.25(d, 2H, *J* = 14.0 Hz, 2CH=), 7.01 (d, 2H, *J* = 8.0 Hz, Ar-H), 6.55 (d, 2H, *J* = 9.0 Hz, Ar-H), 4.50 (d, *J* = 6.4 Hz, 2H, CH_2_), 3.12 (s, 12H, 4CH_3_); ^13^C-NMR (DMSO-d_6_, *δ*, ppm): 168.1, 165.5, 160.6, 151.9, 134.4, 118.7, 145.2, 95.3, 28.1, 27.4; LC/MS (ESI): 455.44 [M + 1]^+^; Anal. Calcd for C_21_H_22_N_6_O_6_: C, 55.50; H, 4.88; N, 18.49; Found: C, 55.65; H, 4.93; N, 18.70.

#### 4.2.17. 5-(((4-(((1,3-Diethyl-4,6-dioxo-2-thioxotetrahydropyrimidin-5(2H)-ylidene)methyl)amino)benzyl)amino)methylene)-1,3-diethyl-2-thioxodihydropyrimidine-4,6(1H,5H)-dione (6)

Compound **6** was synthesised from **2a** (2 equiv.) and 4-aminobenzylamine (1 equiv.) following the general procedure, affording the product as a yellow powder in 86% yield; mp 247 °C; IR (KBr, cm^−1^): 3302, 2958, 2908, 2866, 1614, 1587, 1545, 1504, 1438, 1409; ^1^H-NMR (CDCl_3_, *δ*, ppm): 12.34 (d, 1H, *J* = 13.6 Hz, NH), 10.77 (m, 1H, NH), 8.68 (d, 1H, *J* = 14.0 Hz, CH=), 8.29 (d, 1H, *J* = 13.6 Hz, CH=), 7.39 (m, 4H, Ar-H), 4.65 (d, 2H, *J* = 6.4 Hz, CH_2_), 4.46 (m, 8H, 4CH_2_), 1.35 (m, 12H, 4CH_3_); 13C-NMR (CDCl_3_, *δ*, ppm): 179.0, 178.8, 163.3, 163.2, 161.0, 160.7, 160.4, 152.7, 138.4, 133.6, 129.7, 129.6, 118.9, 118.8, 95.4, 93.6, 53.6, 43.2, 43.0, 42.5, 42.4, 12.5, 12.4, 12.3, 12.2; LC/MS (ESI): 543.67 [M + 1]^+^; Anal. Calcd for C_25_H_30_N_6_O_4_S_2_: C, 55.33; H, 5.57; N, 15.49; Found: C, 55.54; H, 5.69; N, 15.66.

### 4.3. Protocol for *in vitro α*-glucosidase inhibition assay

The assay protocol for was performed spectrophotometrically following the reported method^22^, where *α*-glucosidase from *S. cerevisiae* (G0660-750UN, Sigma Aldrich) was dissolved in phosphate buffer (pH 6.8, 50 mM). Test compounds were dissolved in 70% DMSO. 20 μL of test sample, 20 μL of enzyme and 135 μL of buffer were added to 96-well plates and incubated for 15 min at 37 °C. After incubation, 25 μL of *p*-nitrophenyl-α-d-glucopyranoside (0.7 mM, Sigma Aldrich) was added and changes in absorbance were monitored for 30 min at 400 nm. The test compound was replaced by DMSO (7.5% final) as control. Acarbose (Acarbose, Sigma Aldrich) was used as a standard inhibitor.

### 4.4. Protocol for anti-glycation assay^40^^,^^41^

The assay was performed following *Gutierrez. R. M. P*, with slight modifications. In brief, Bovine Serum Albumin solution (10 mg/mL) was prepared in 100 mM of phosphate buffer pH 7.4 containing 3 mM sodium azide as antimicrobial agent. A methylglyoxal solution of 14 mM was also prepared in the same buffer. 1-mM concentrations of the test compounds and standard inhibitor were prepared in dimethyl sulfoxide (DMSO). Each well of a 96-well plate contained 20 µL of inhibitor, 50 µL of BSA, 50 µL of methylglyoxal and 80 µL of phosphate buffer, while the control contained 20 µl of DMSO instead of test compound. The total reaction volume was 200 µL. The reaction mixture was then incubated for 9 days at 37 °^ ^C. After incubation, each sample was examined for the development of specific fluorescence (excitation 330 nm; emission 420 nm) against a blank on a microplate reader (Spectramax M2 Devices, CA, USA).

### 4.5. Calculation of inhibitory activity

The percentage inhibition of advanced glycation end (AGEs) products formation by the test sample versus control was calculated using the following formula:

$$\text{The}\;\%\;\text{inhibition of AGE formation}=1-\left[\left(\frac{\text{fluorescence of the test group}}{\text{fluorescence of the control group}}\right)\right]\times 100\%$$

## Supplementary Material

Supplemental Material
