# Supplementary material for: Synthesis and characterisation of thiobarbituric acid enamine derivatives, and evaluation of their α-glucosidase inhibitory and anti-glycation activity
Source: J Enzyme Inhib Med Chem. 2020 Mar 11;35(1):692–701. doi: 10.1080/14756366.2020.1737045 (PMC7155210; doi:10.1080/14756366.2020.1737045)

Synthesis and characterization of thiobarbituric acid enamine derivatives, and evaluation of their α-glucosidase inhibitory and anti-glycation activity

M. Ali, Assem Barakat,* Ayman El-Faham*, Hessa H. Al-Rasheed, Kholoud Dahlous, Abdullah Mohammed Al-Majid , Anamika Sharma, Sammer Yousuf, Mehar Sanam, Zaheer Ul-Haq, M. Iqbal Choudhary, Beatriz G. de la Torre, Fernando Albericio

Table of content

**Figure S1**	^{1}HNMR and ^{13}CNMR ($CDCl_3$) **3a**
**Figure S2**	^{1}HNMR and ^{13}CNMR ($CDCl_3$) **3b**
**Figure S3**	^{1}HNMR and ^{13}CNMR ($CDCl_3$) **3c**
**Figure S4**	^{1}HNMR ($CDCl_3$) and ^{13}CNMR (DMSO-d_6) **3d**
**Figure S5**	^{1}HNMR and ^{13}CNMR ($CDCl_3$) **3e**
**Figure S6**	^{1}HNMR and ^{13}CNMR ($CDCl_3$) **3f**
**Figure S7**	^{1}HNMR and ^{13}CNMR ($CDCl_3$) **3g**
**Figure S8**	^{1}HNMR and ^{13}CNMR ($CDCl_3$) **3h**
**Figure S9**	^{1}HNMR and ^{13}CNMR ($CDCl_3$) **3i**
**Figure S10**	^{1}HNMR ($CDCl_3$) and ^{13}CNMR (DMSO-d_6) **3j**
**Figure S11**	^{1}HNMR and ^{13}CNMR ($CDCl_3$) **4a**
**Figure S12**	^{1}HNMR and ^{13}CNMR ($CDCl_3$) **4b**
**Figure S13**	^{1}HNMR and ^{13}CNMR ($CDCl_3$) **4c**
**Figure S14**	^{1}HNMR and ^{13}CNMR ($CDCl_3$) **4d**
**Figure S15**	^{1}HNMR ($CDCl_3$) and ^{13}CNMR (DMSO-d_6) **5**
**Figure S16**	^{1}HNMR ($CDCl_3$) and ^{13}CNMR (DMSO-d_6) **6**

Figure S1: ^{1}HNMR and ^{13}CNMR ($CDCl_3$) **3a**

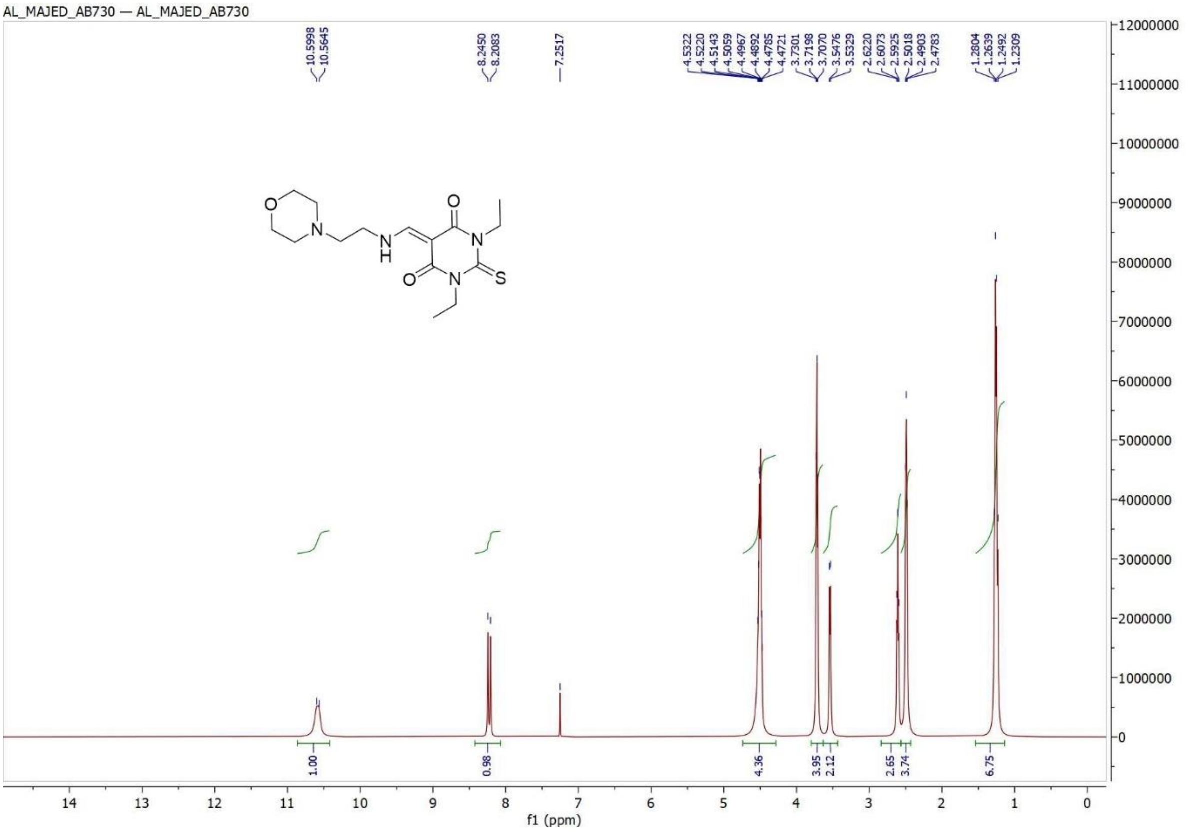

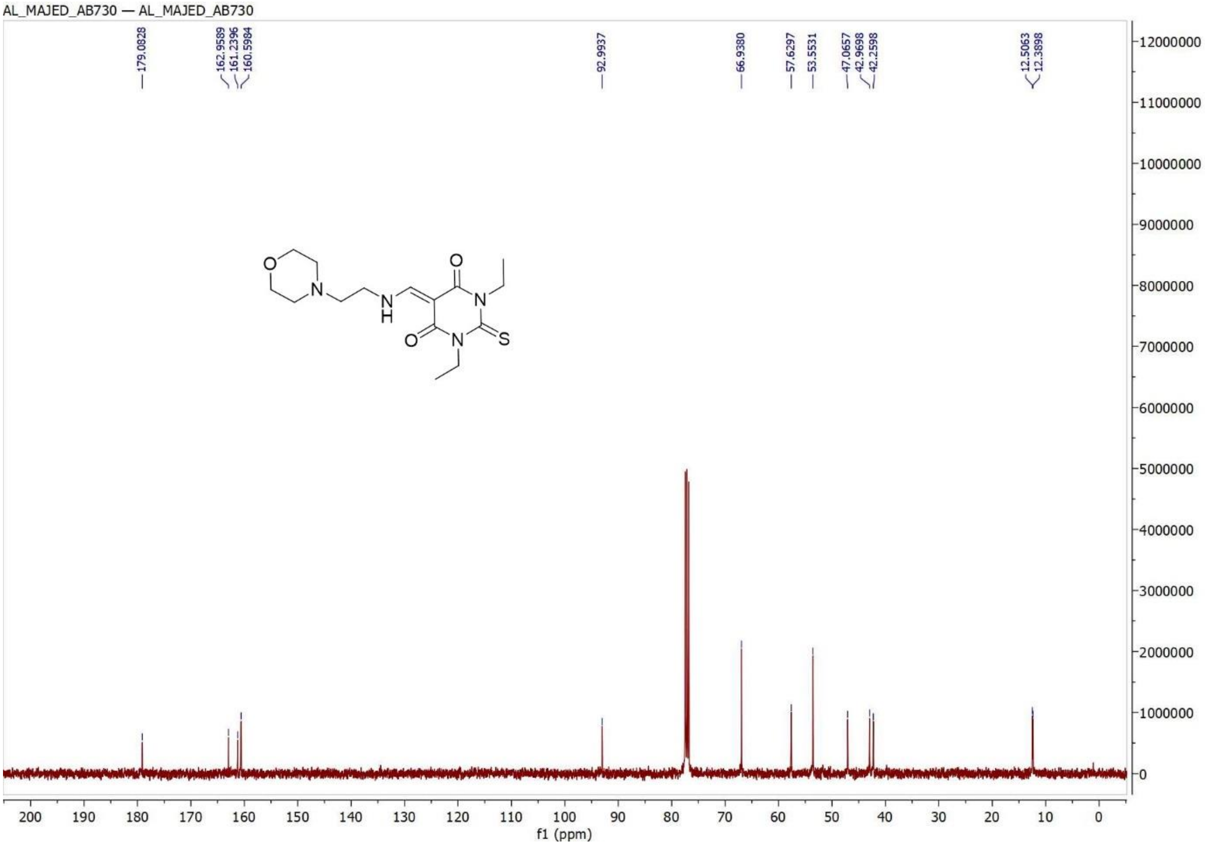

Figure S2: ^{1}HNMR and ^{13}CNMR ($CDCl_3$) **3b**

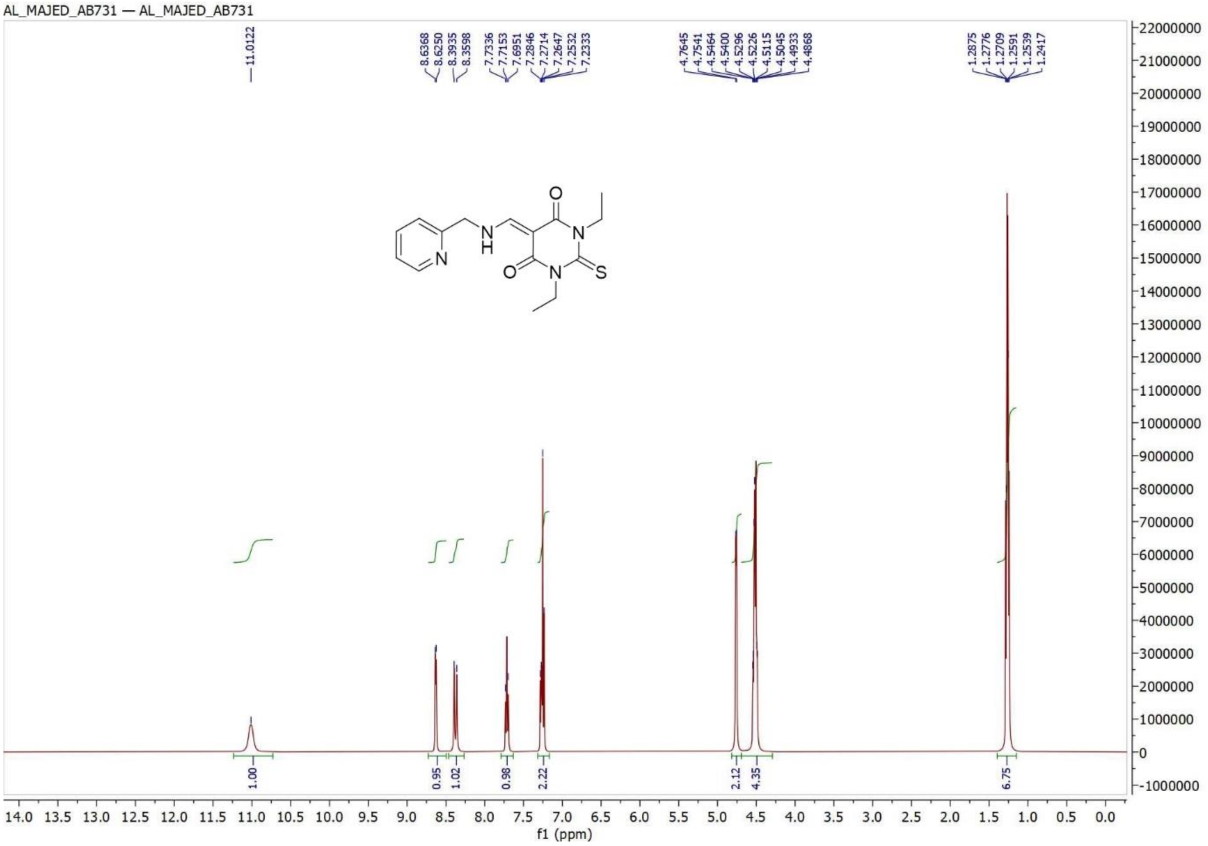

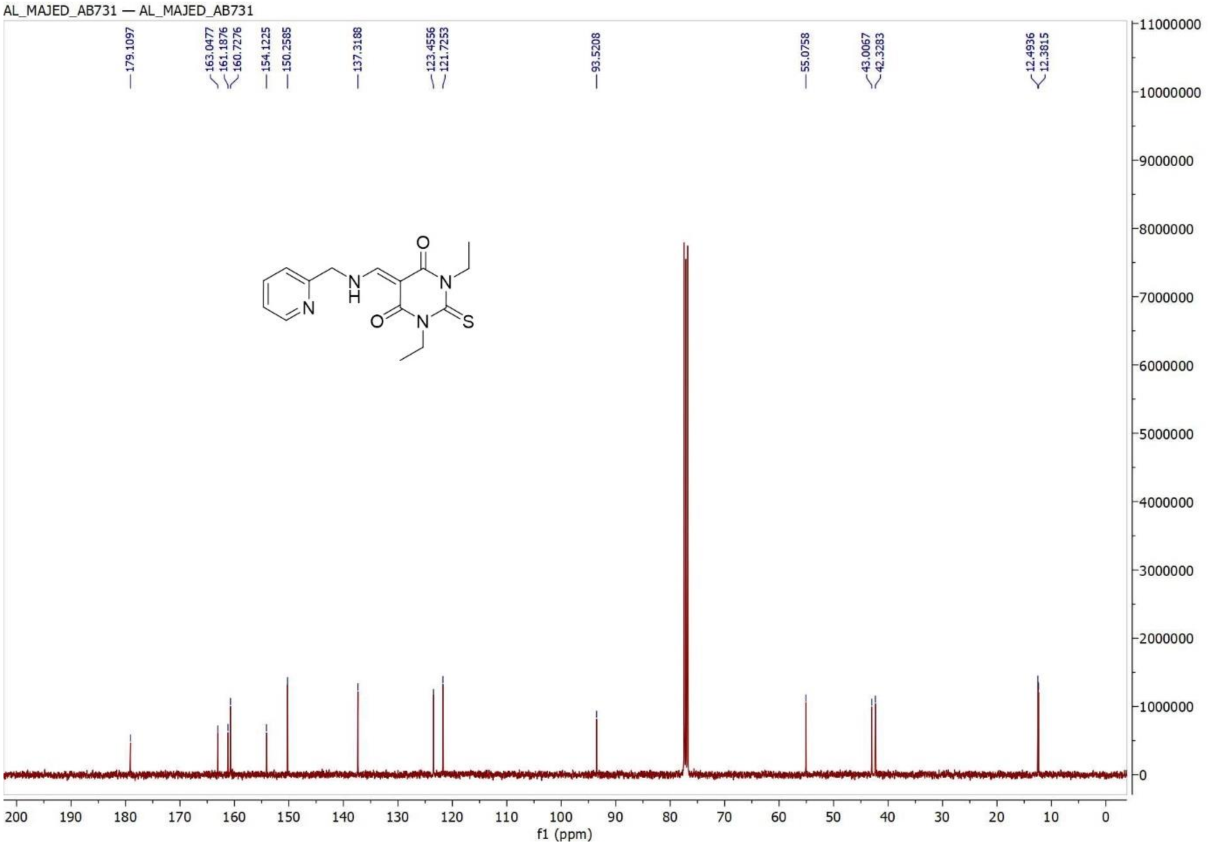

Figure S3: ^{1}HNMR and ^{13}CNMR ($CDCl_3$) **3c**

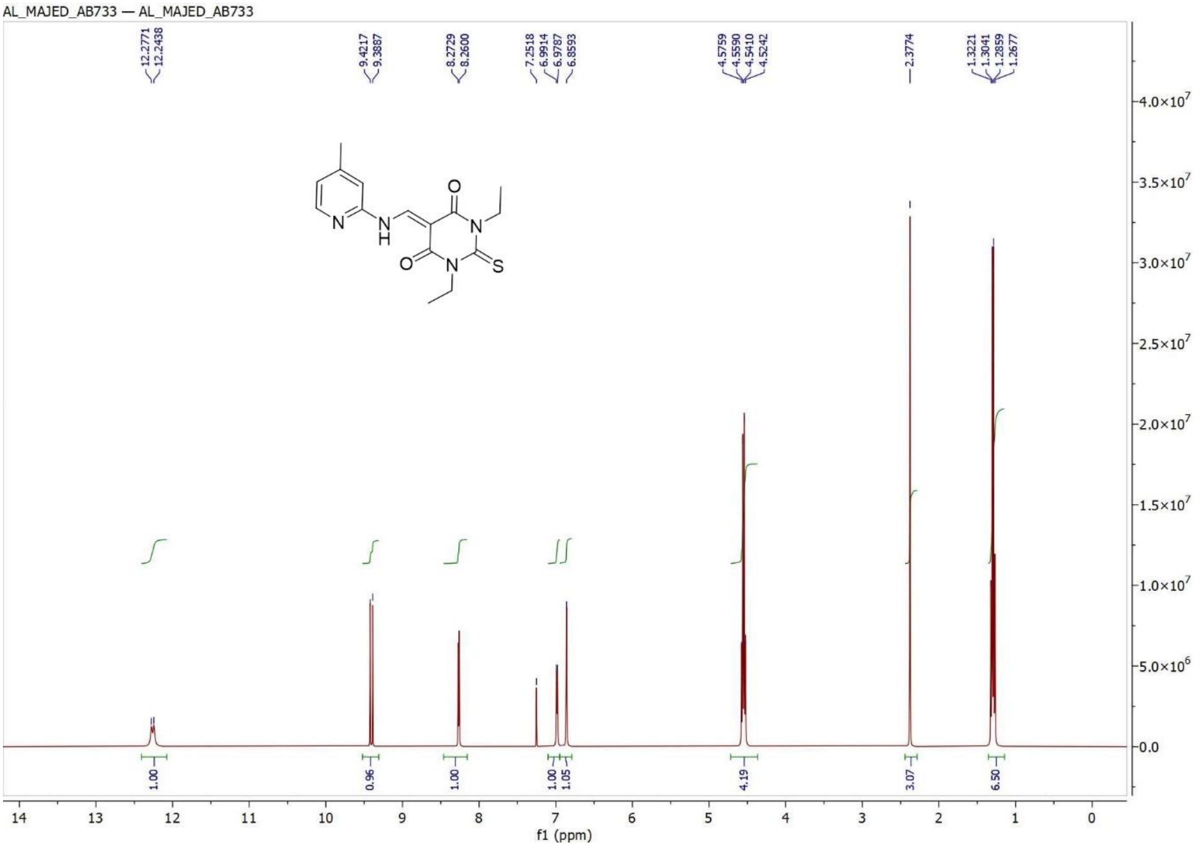

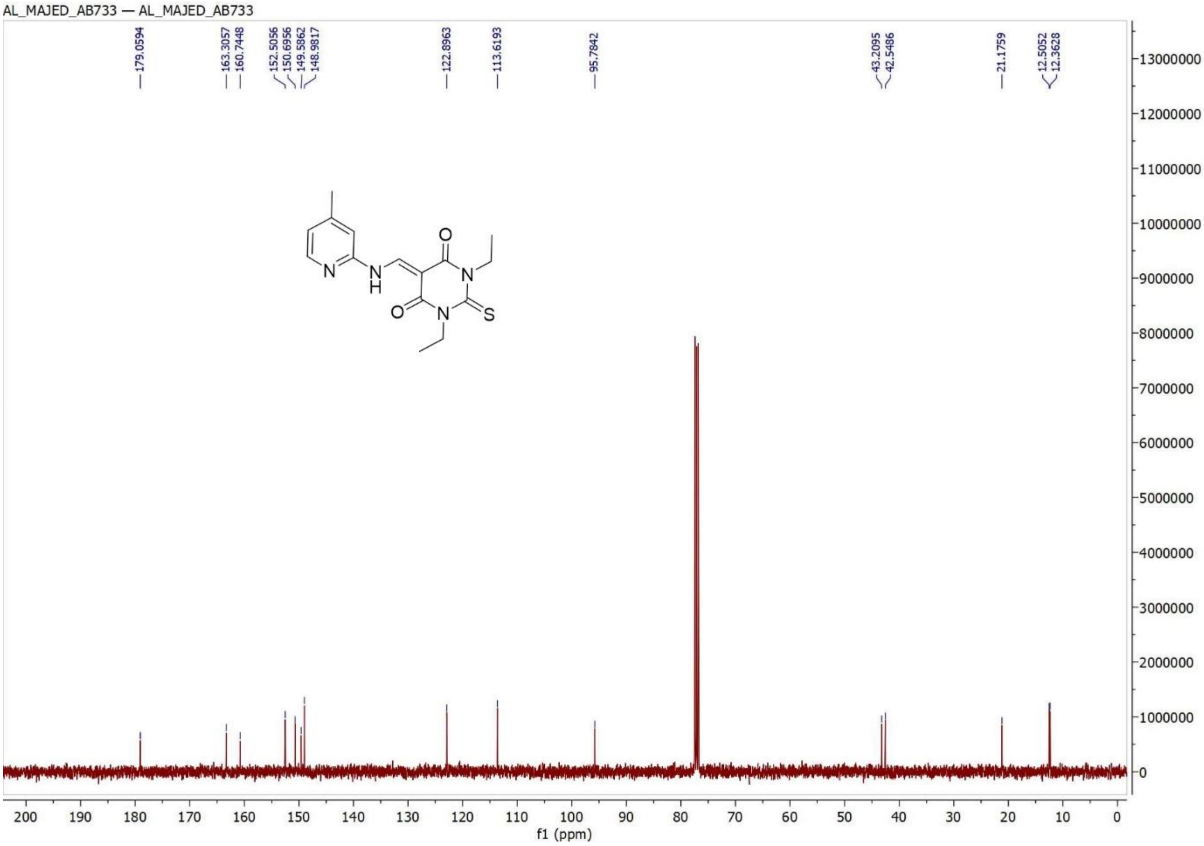

Figure S4: ^{1}HNMR ($CDCl_3$) and ^{13}CNMR (DMSO-d_6) **3d**

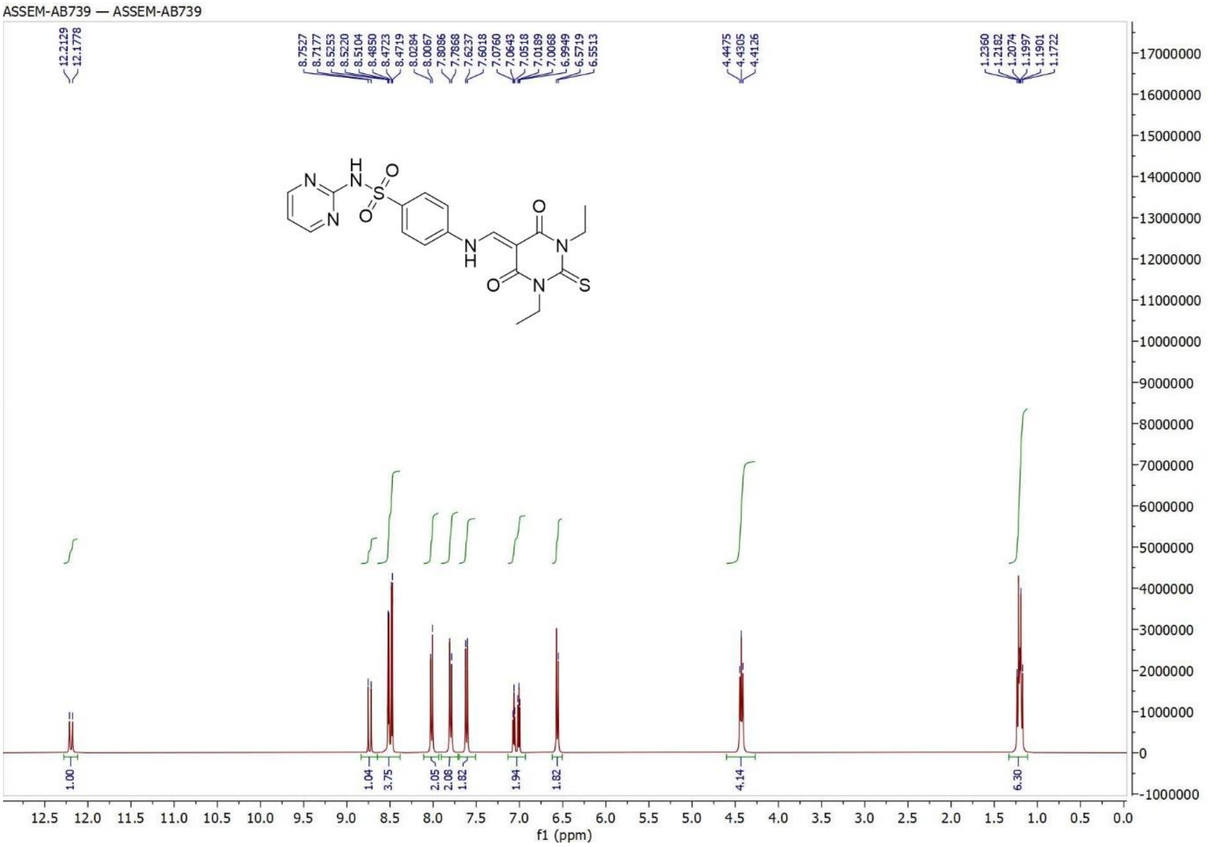

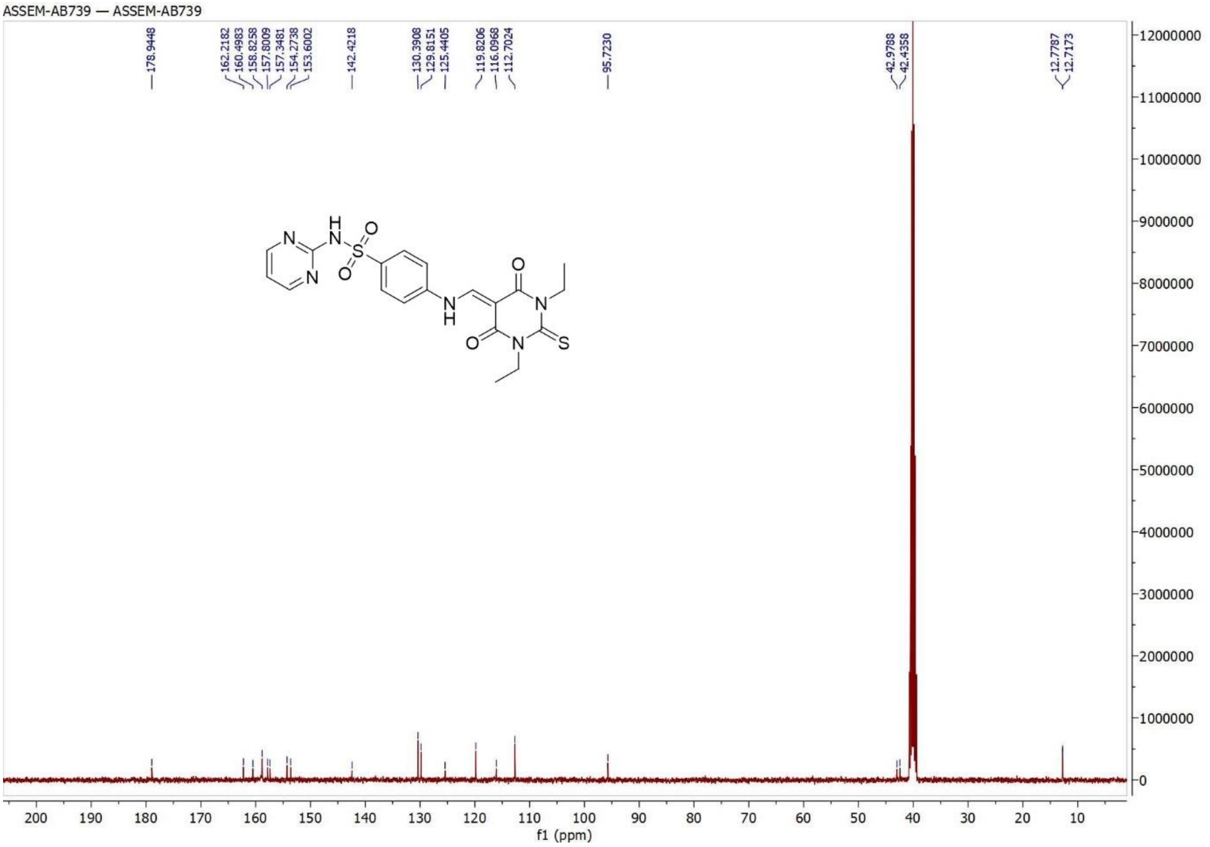

Figure S5: ^{1}HNMR and ^{13}CNMR ($CDCl_3$) **3e**

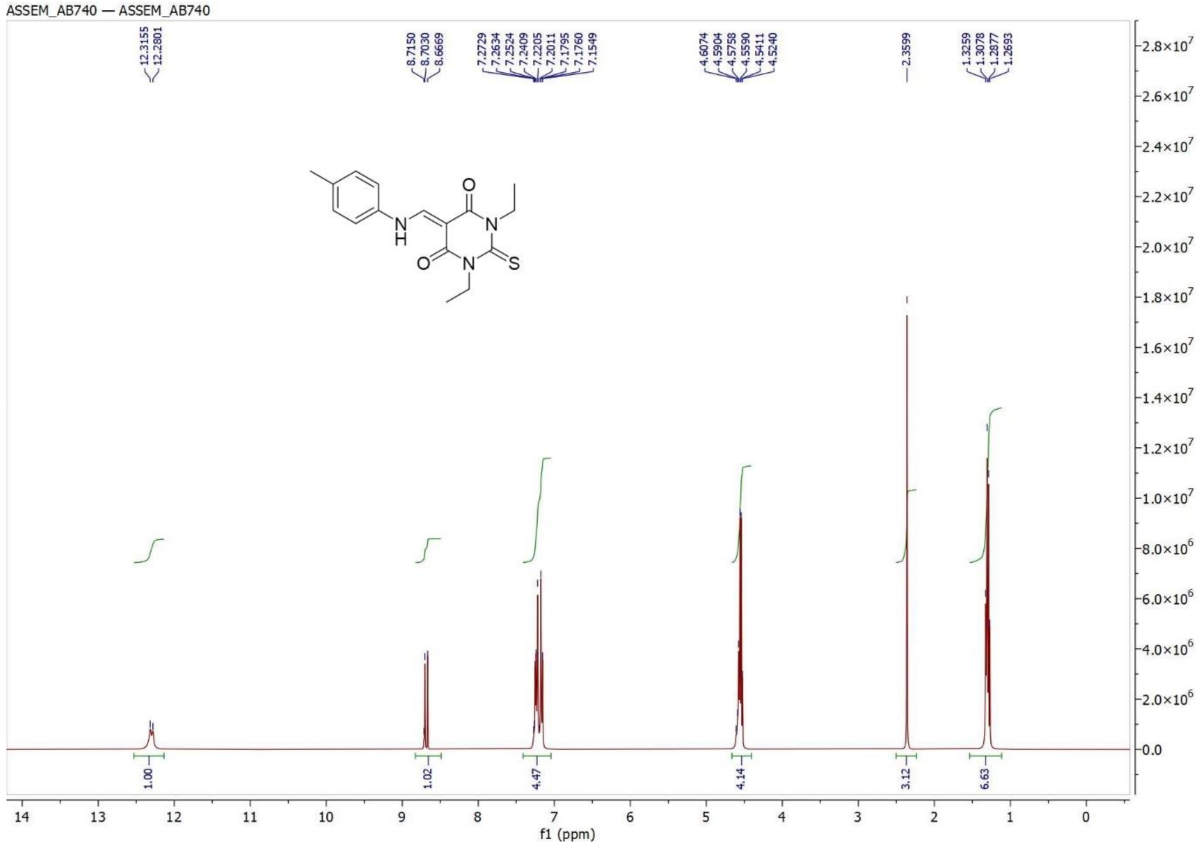

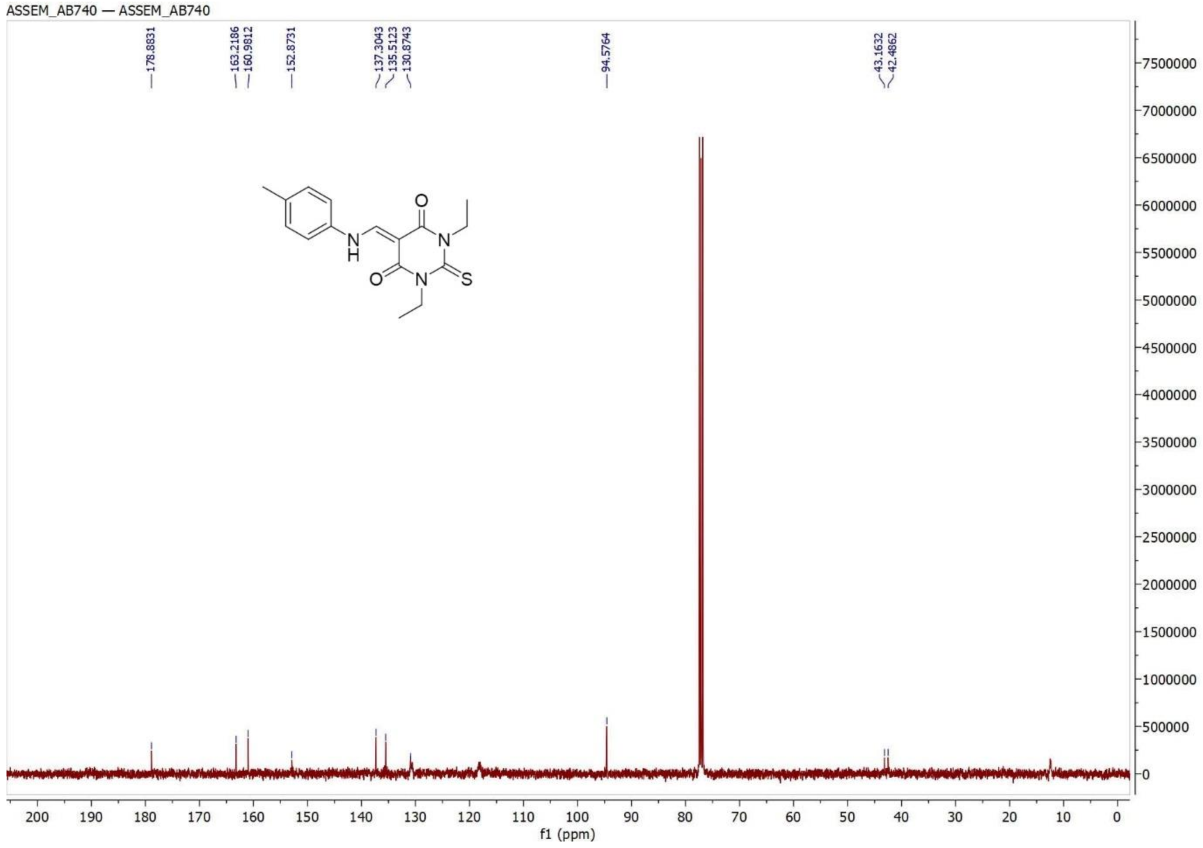

Figure S6: ^{1}HNMR and ^{13}CNMR ($CDCl_3$) **3f**

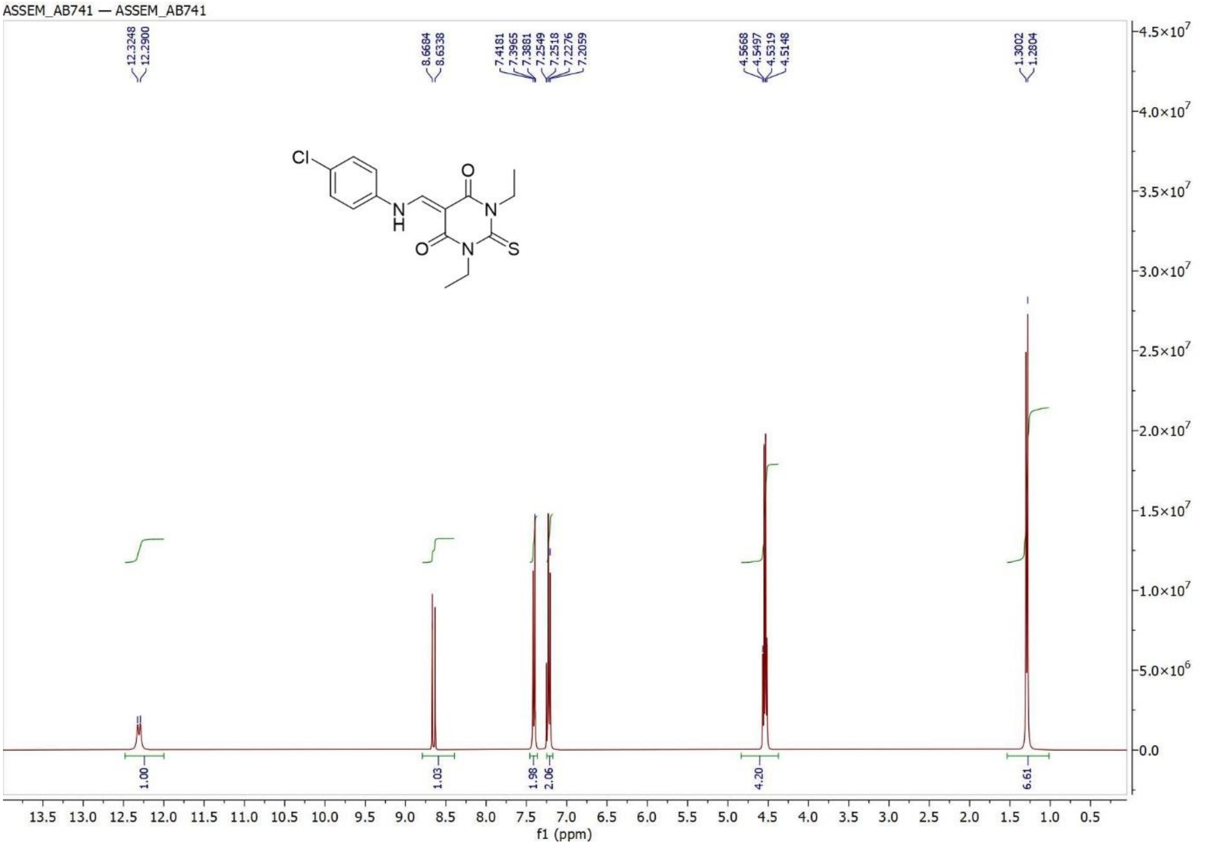

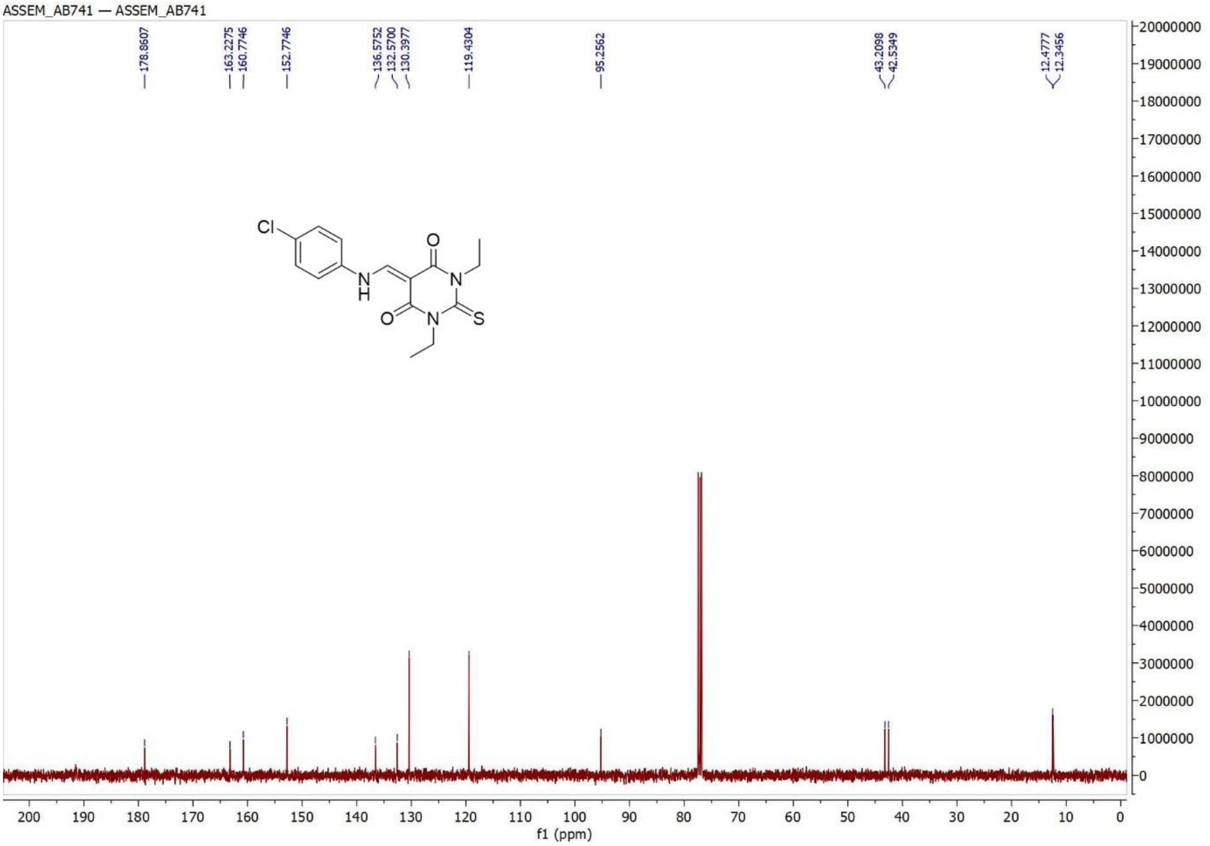

Figure S7: ^{1}HNMR and ^{13}CNMR ($CDCl_3$) **3g**

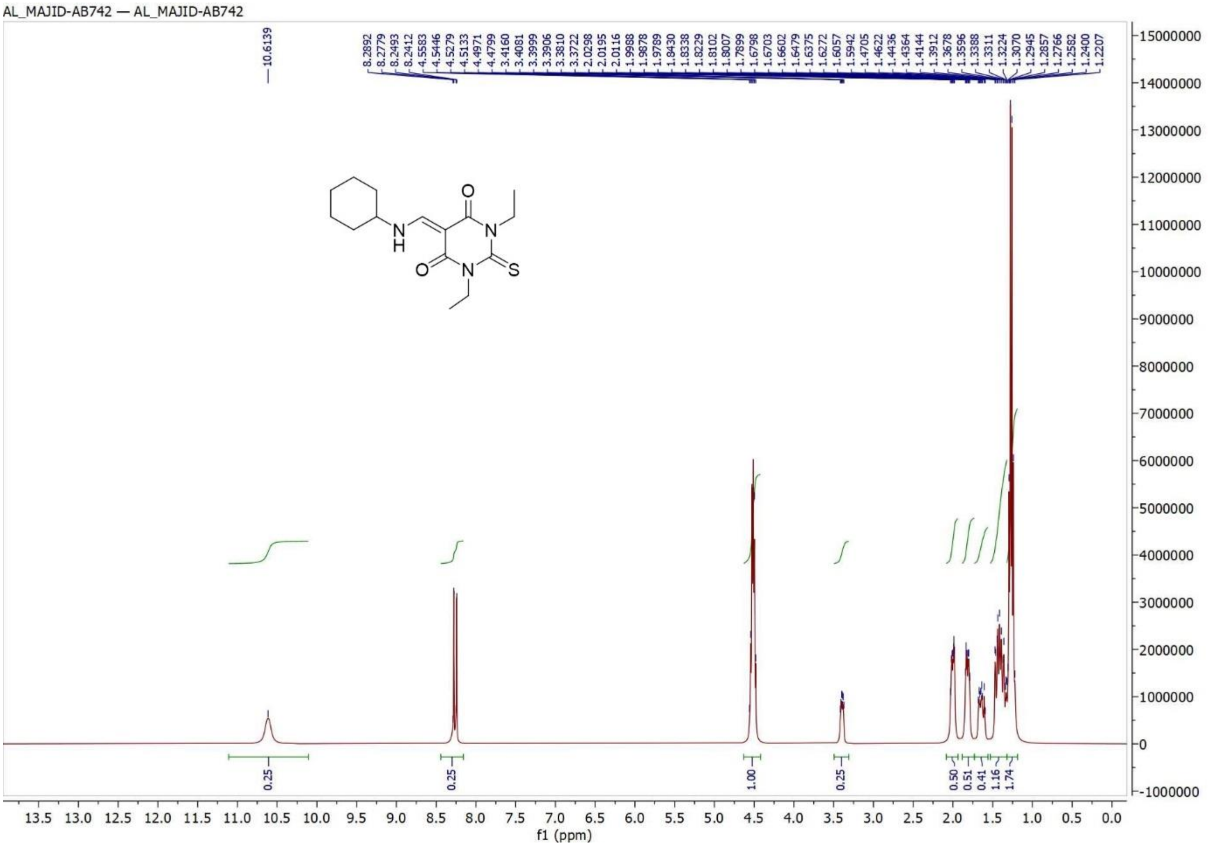

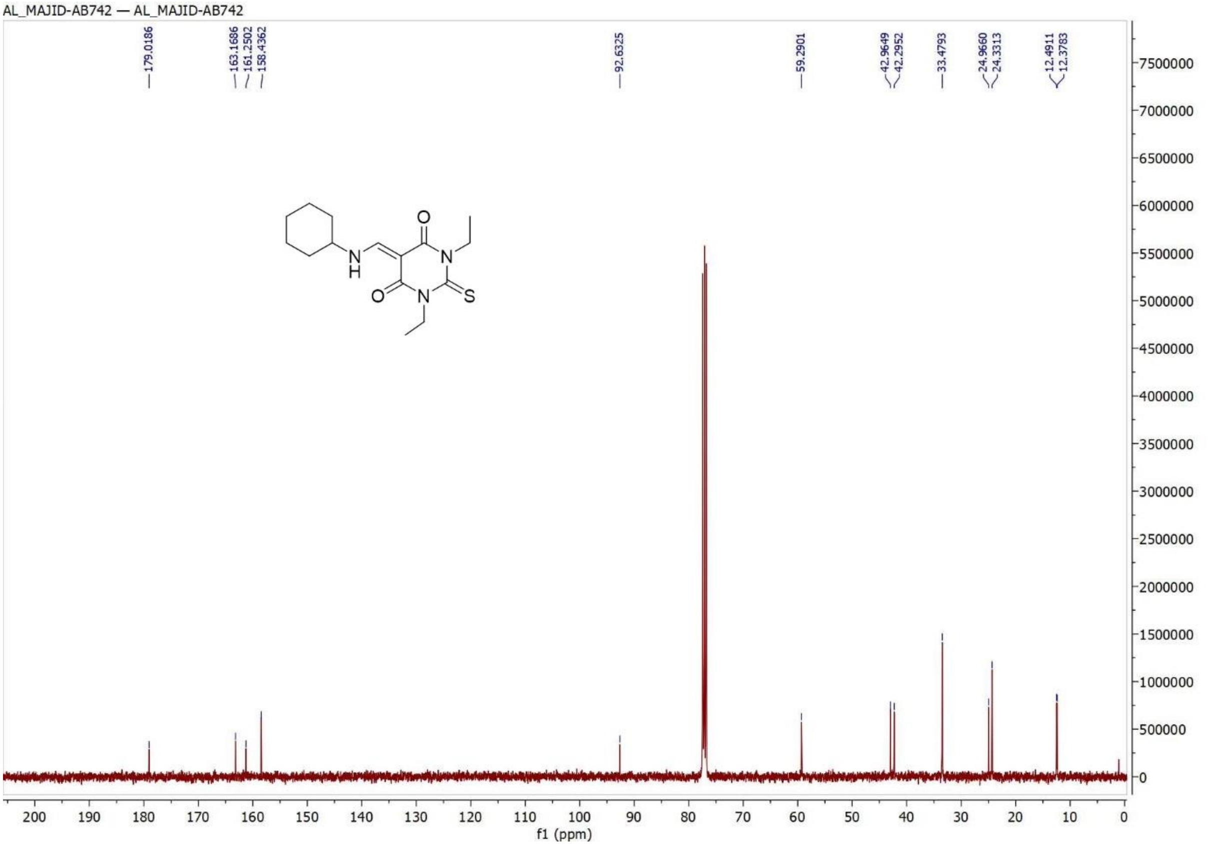

Figure S8: ^{1}HNMR and ^{13}CNMR ($CDCl_3$) **3h**

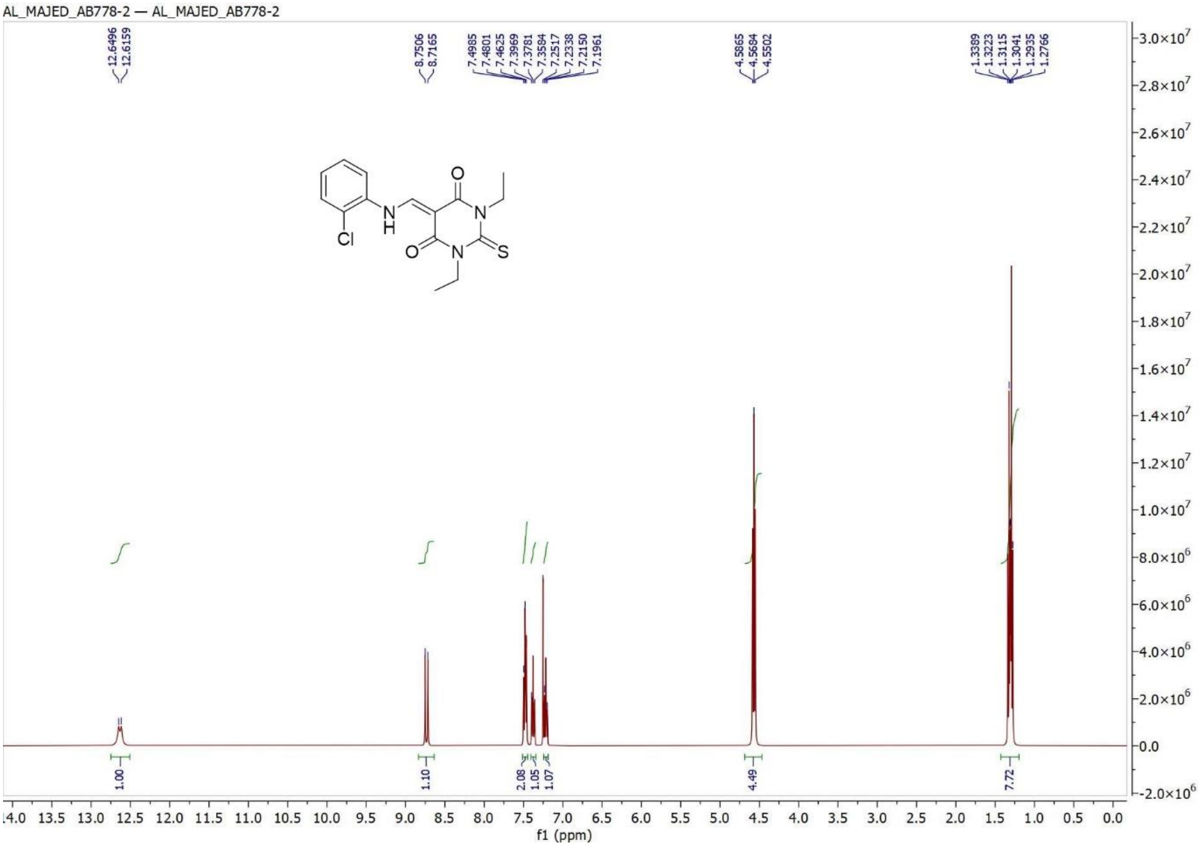

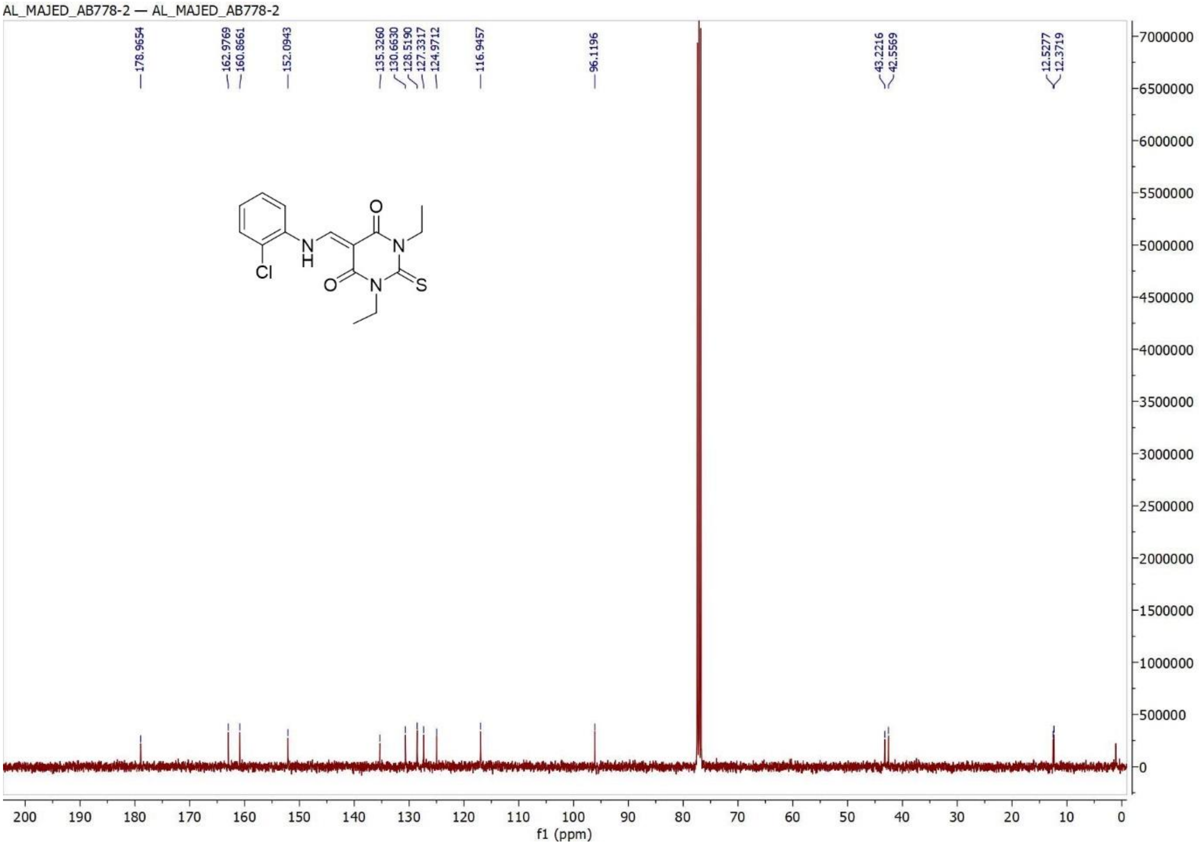

Figure S9: ^{1}HNMR and ^{13}CNMR ($CDCl_3$) **3i**

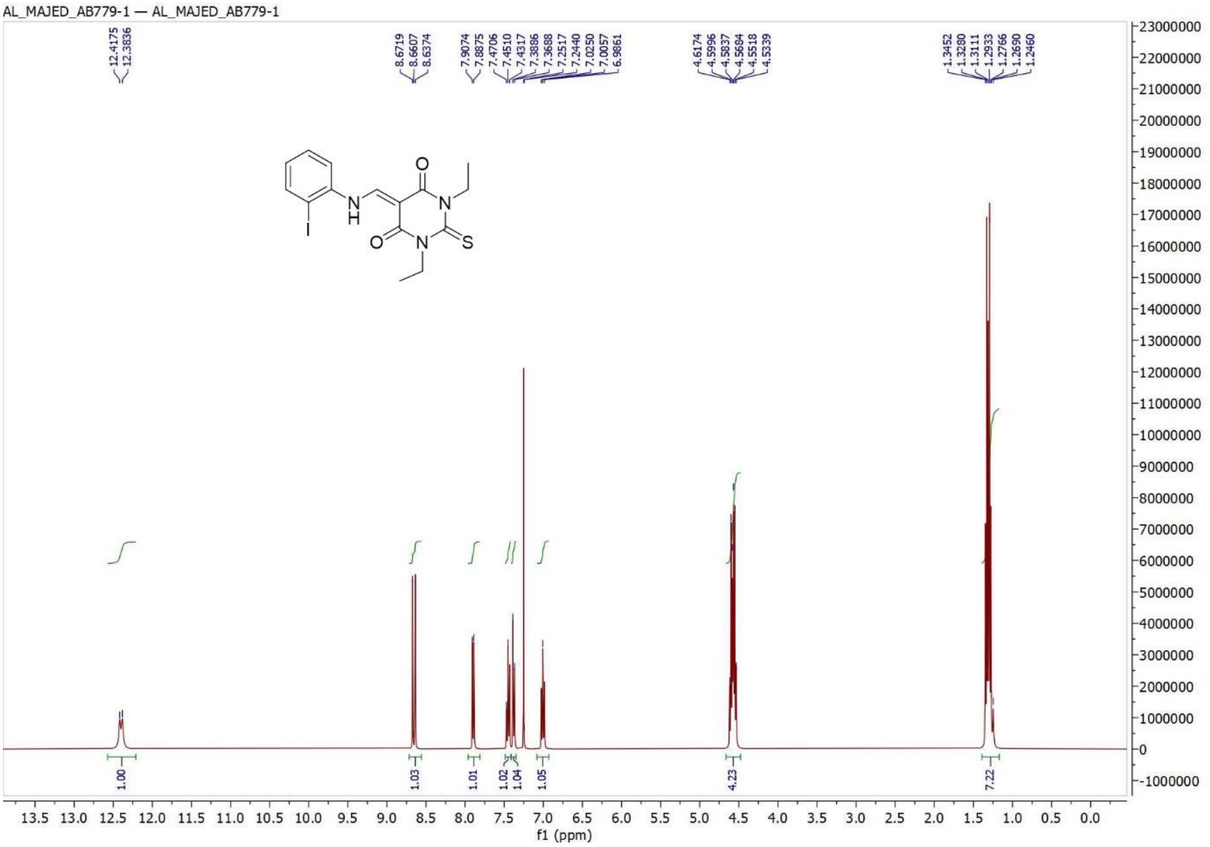

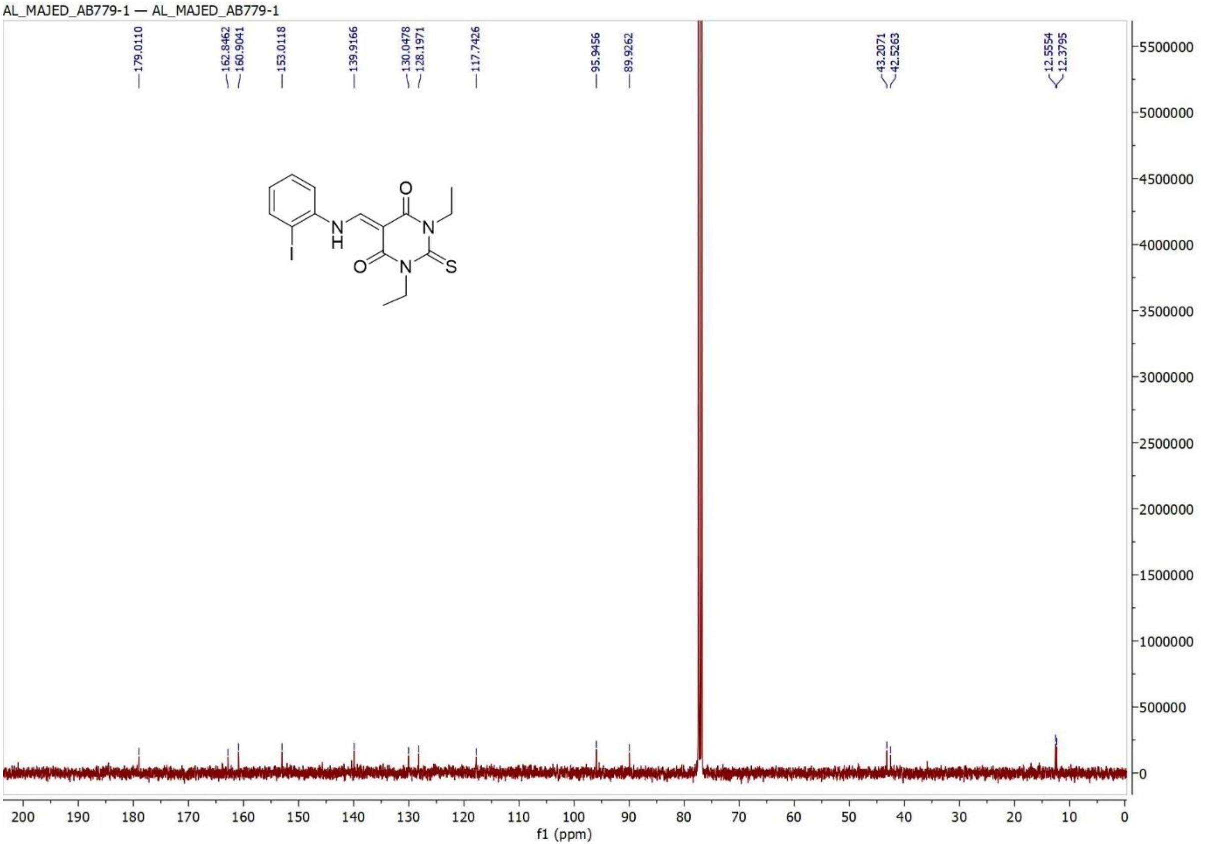

Figure S10: ^{1}HNMR ($CDCl_3$) and ^{13}CNMR (DMSO-d_6) **3j**

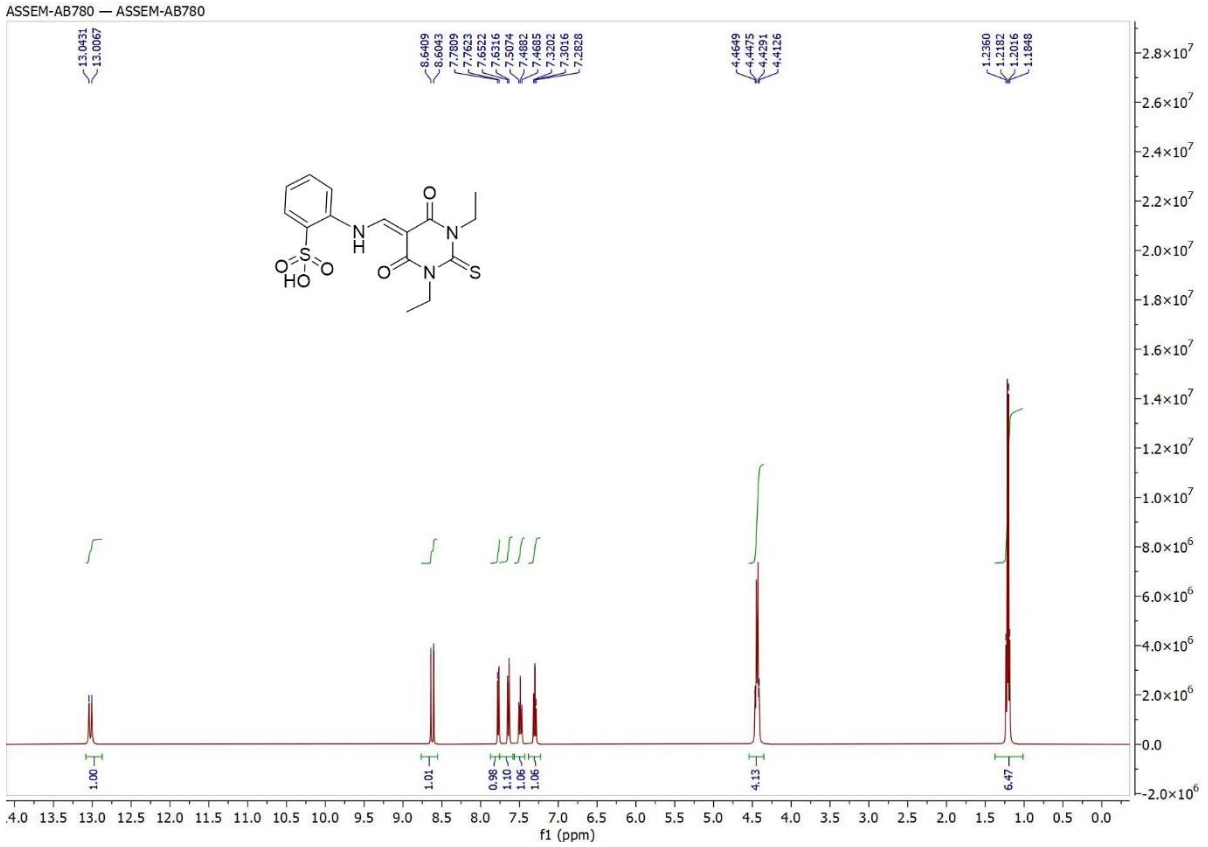

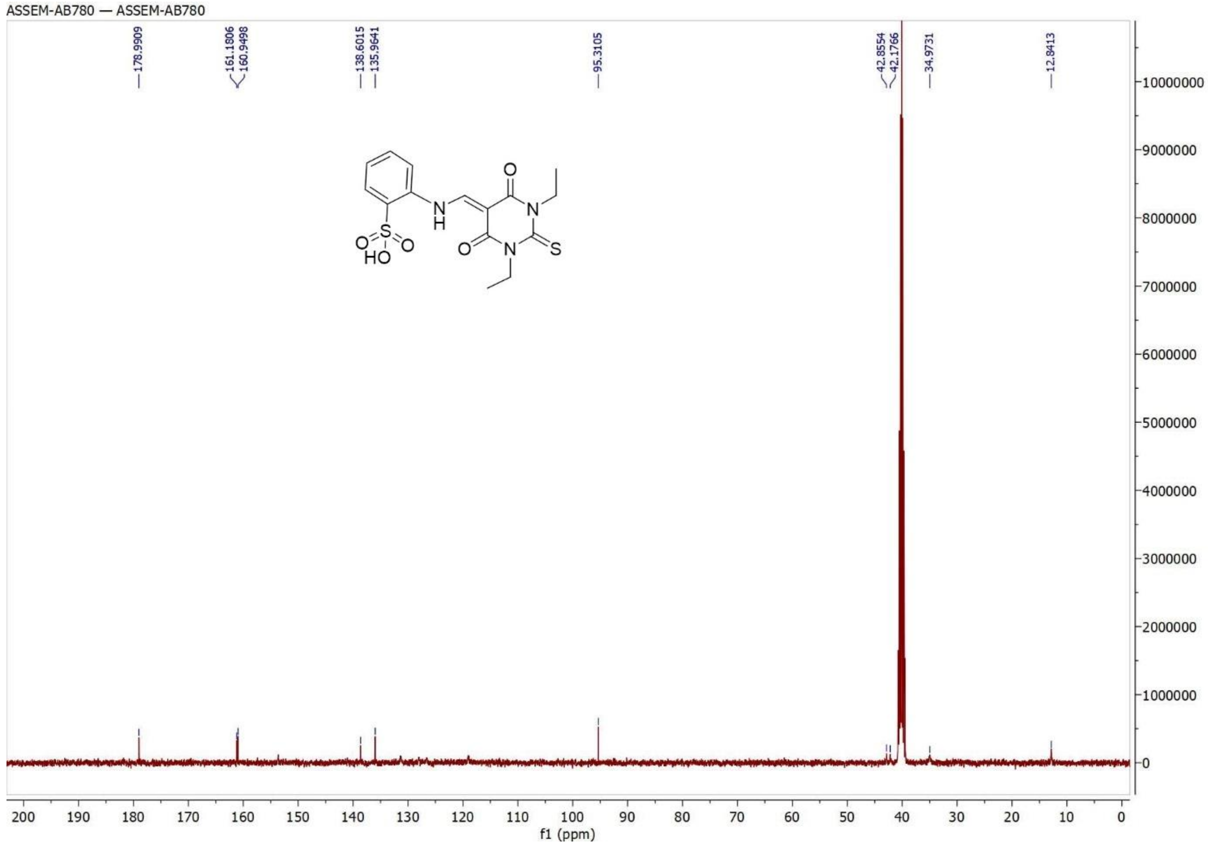

Figure S11: ^{1}HNMR and ^{13}CNMR ($CDCl_3$) **4a**

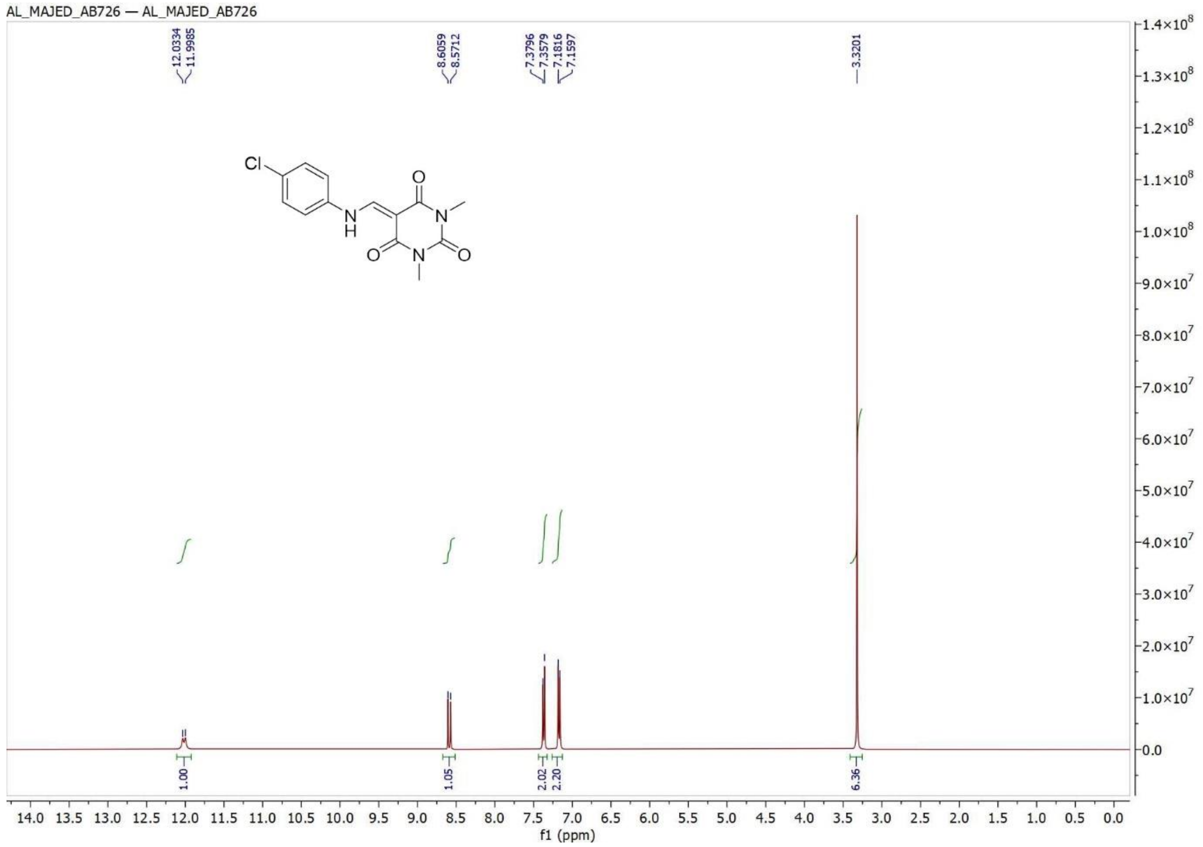

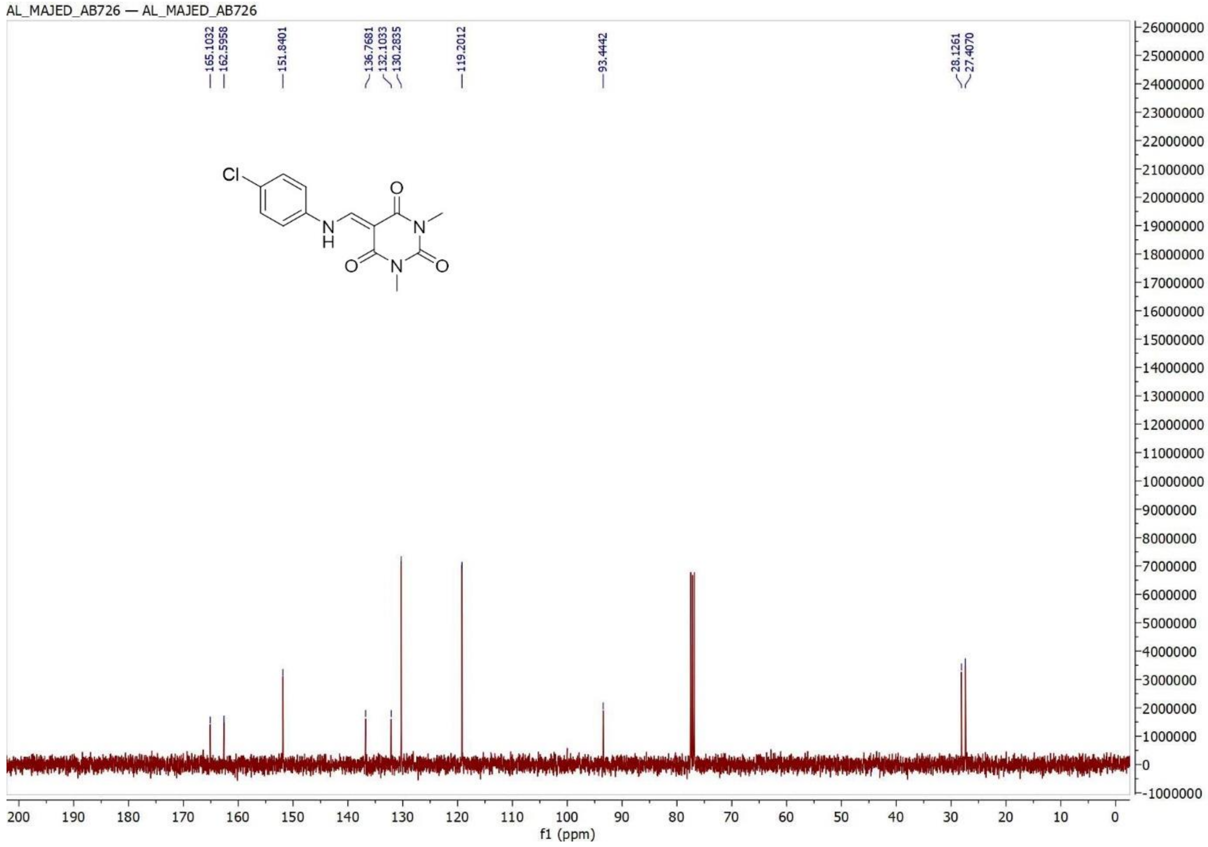

Figure S12: ^{1}HNMR and ^{13}CNMR ($CDCl_3$) **4b**

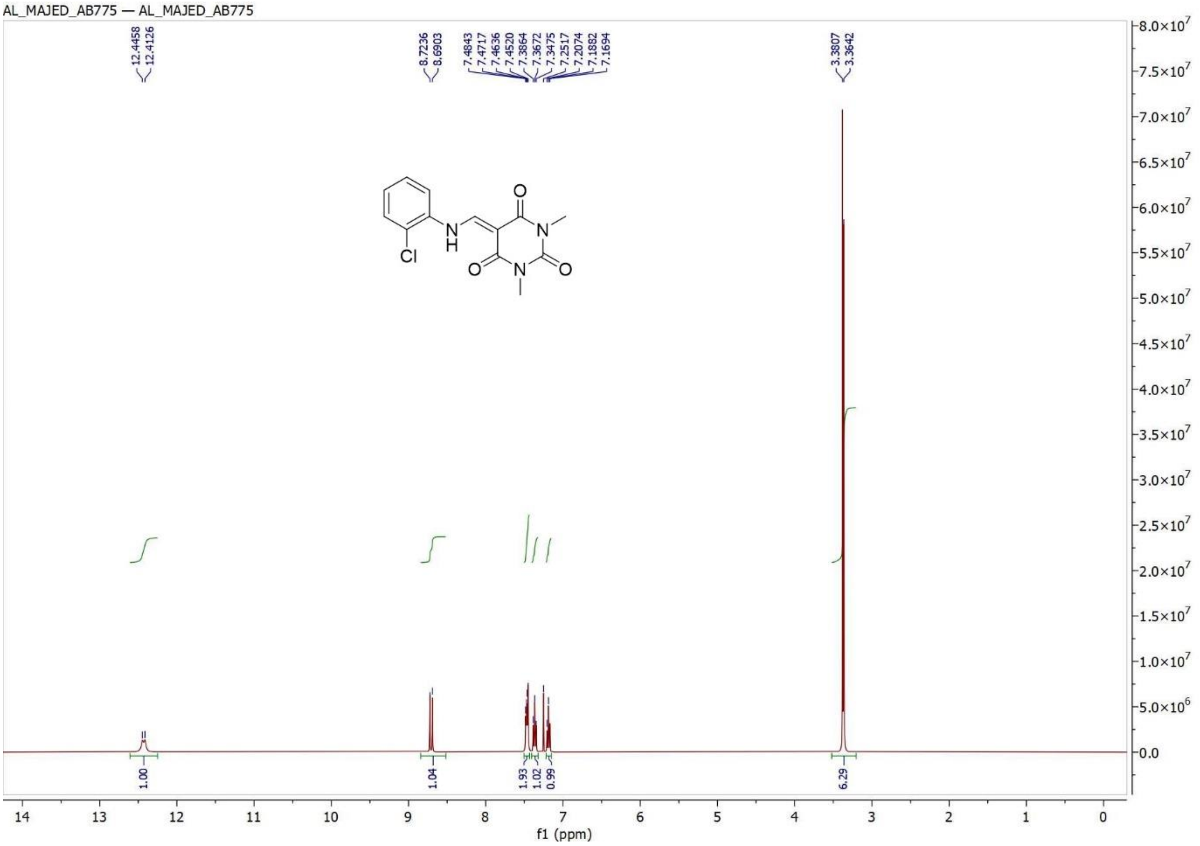

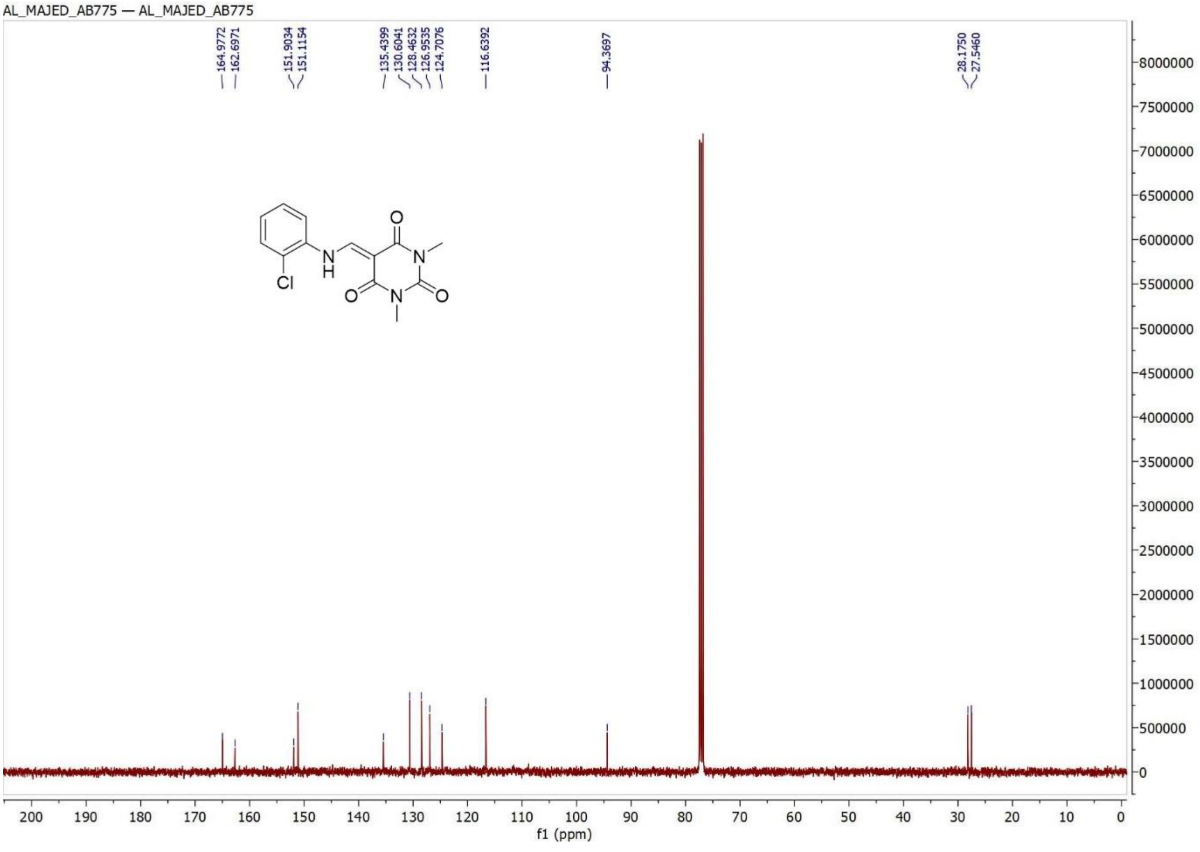

Figure S13: ^{1}HNMR and ^{13}CNMR ($CDCl_3$) **4c**

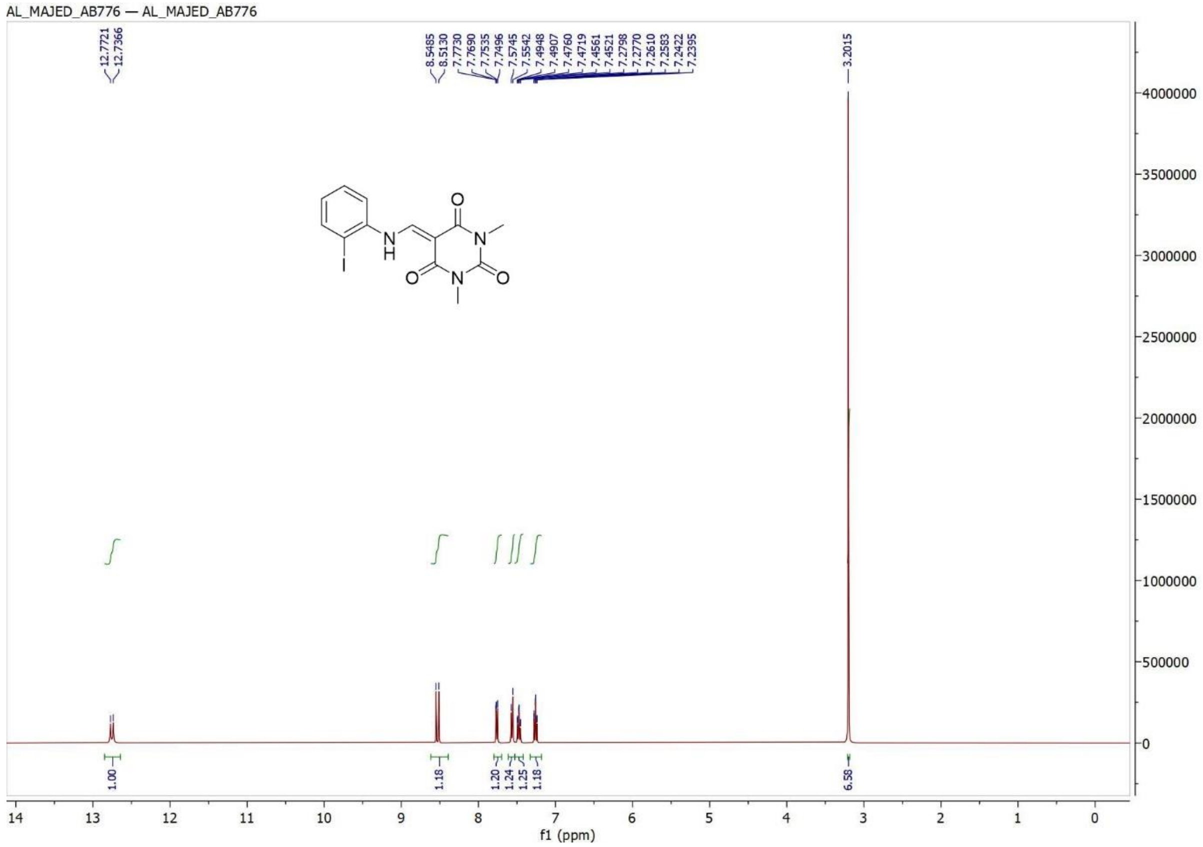

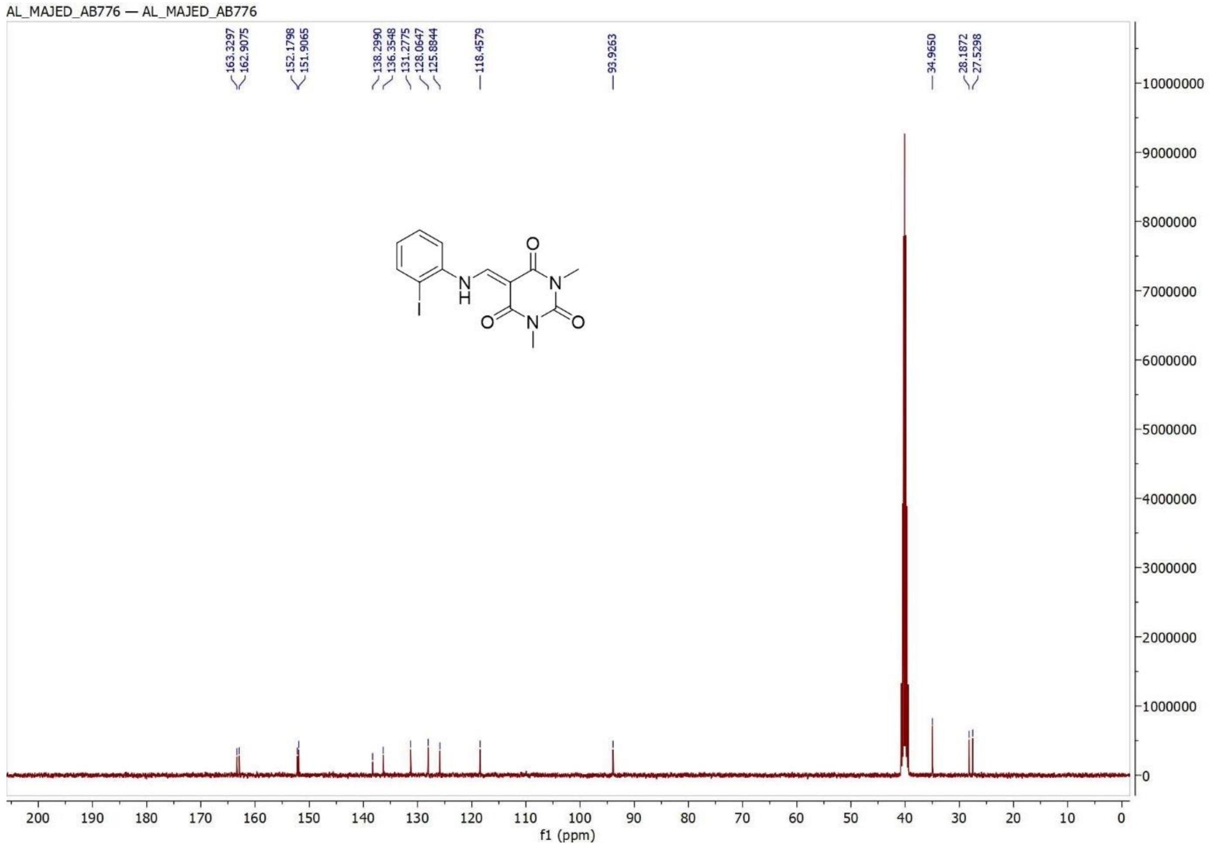

Figure S14: ^{1}HNMR and ^{13}CNMR ($CDCl_3$) **4d**

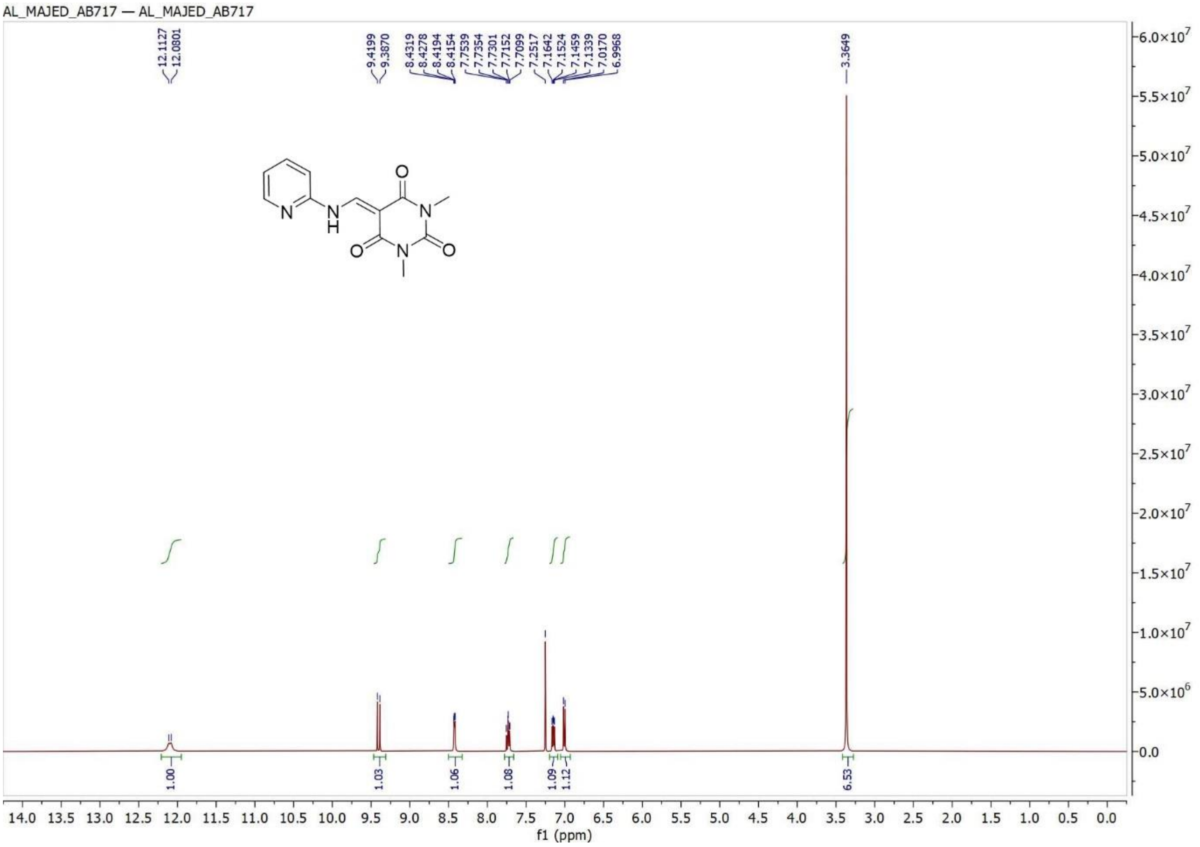

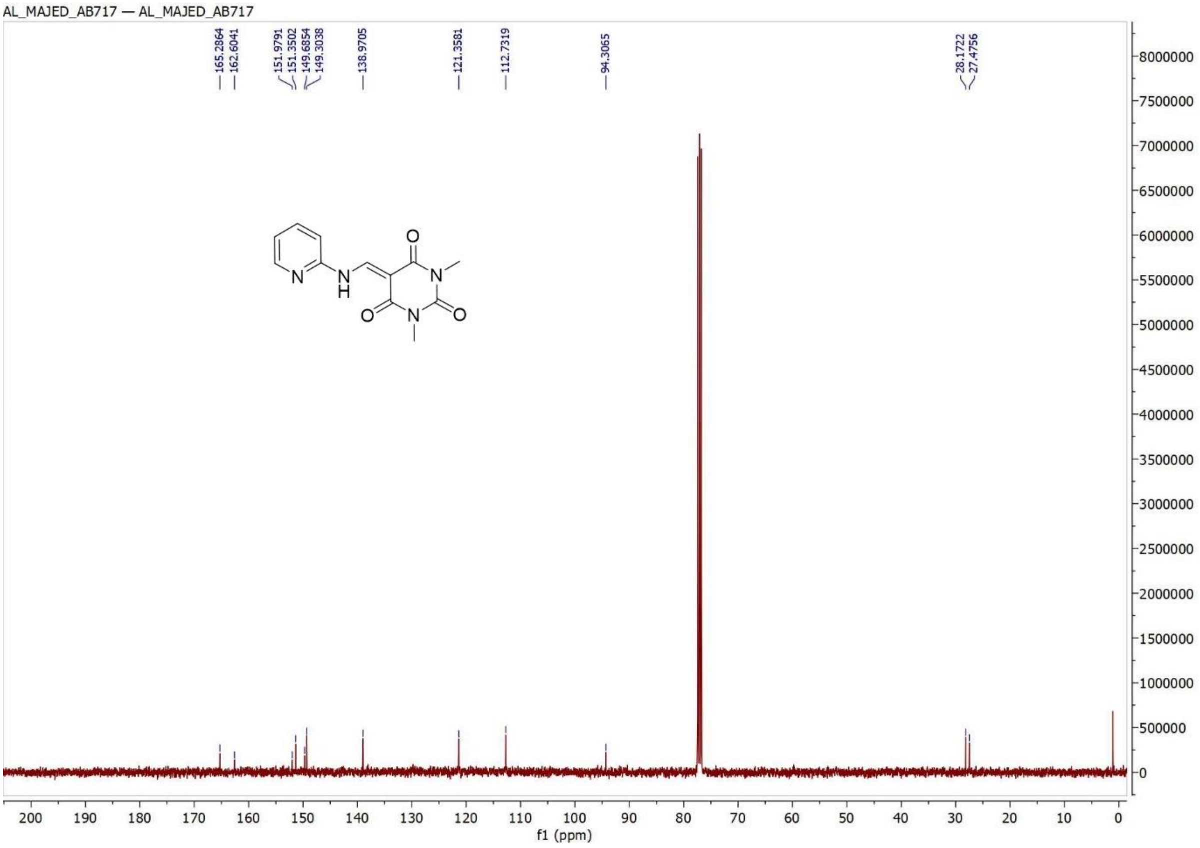

Figure S15: ^{1}HNMR ($CDCl_3$) and ^{13}CNMR (DMSO-d_6) **5**

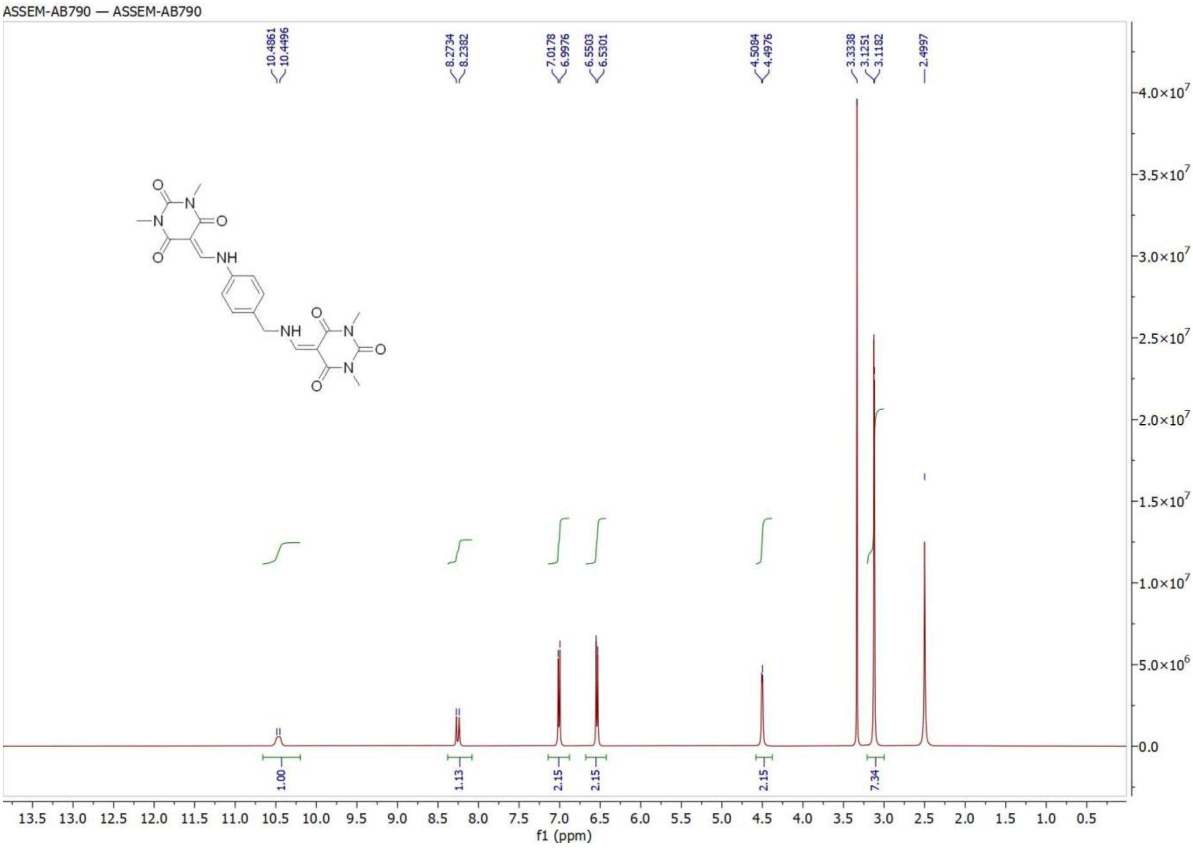

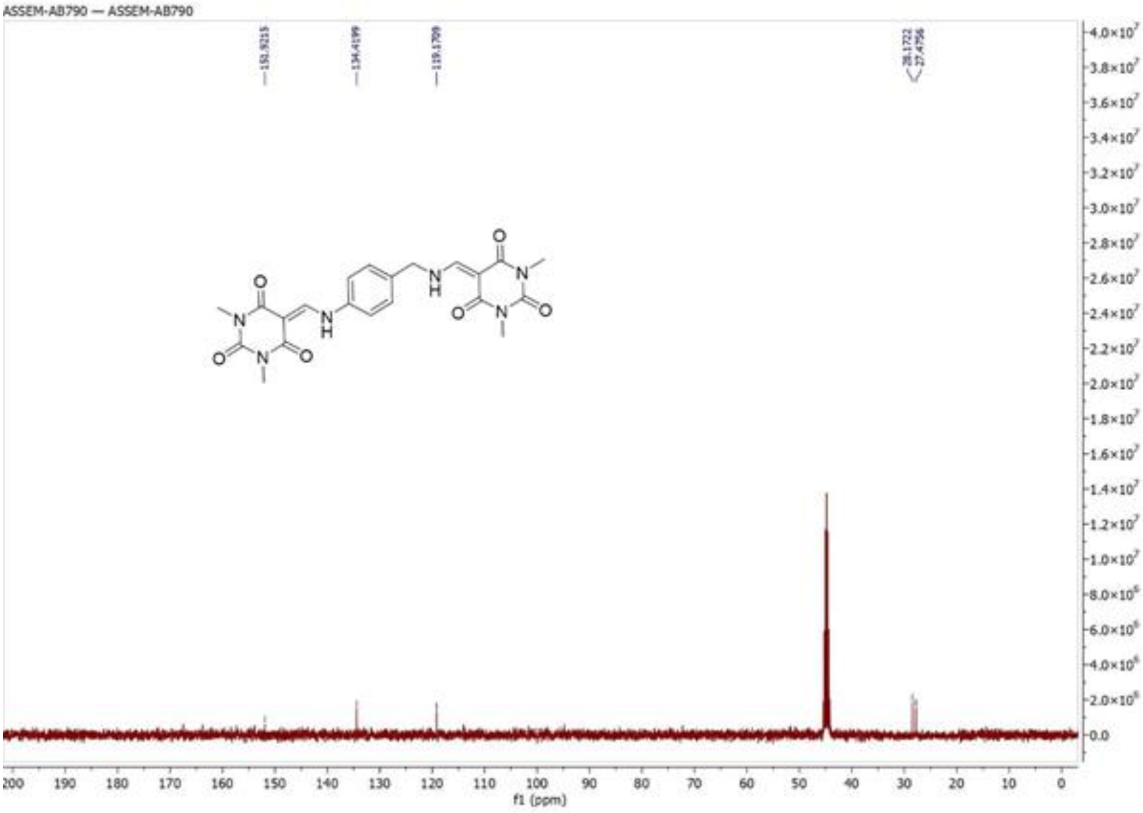

Figure S16: ^{1}HNMR ($CDCl_3$) and ^{13}CNMR (DMSO-d_6) **6**

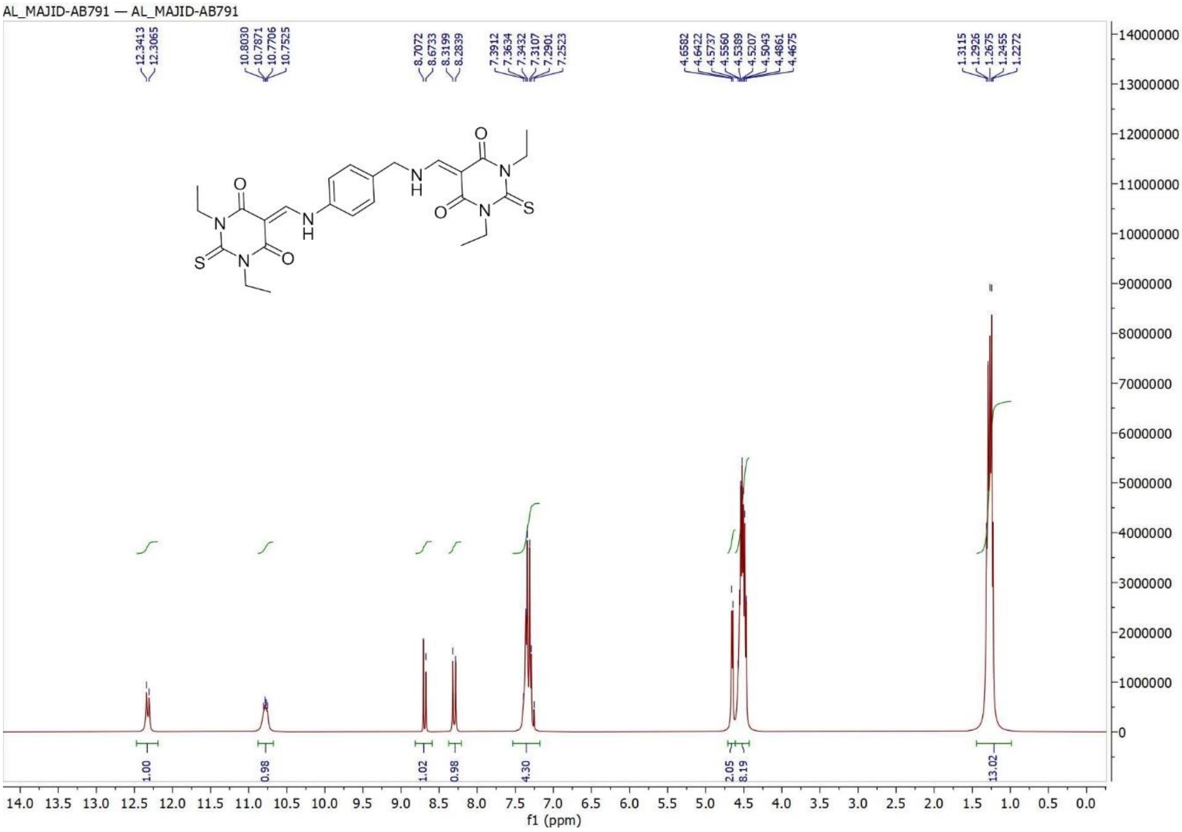

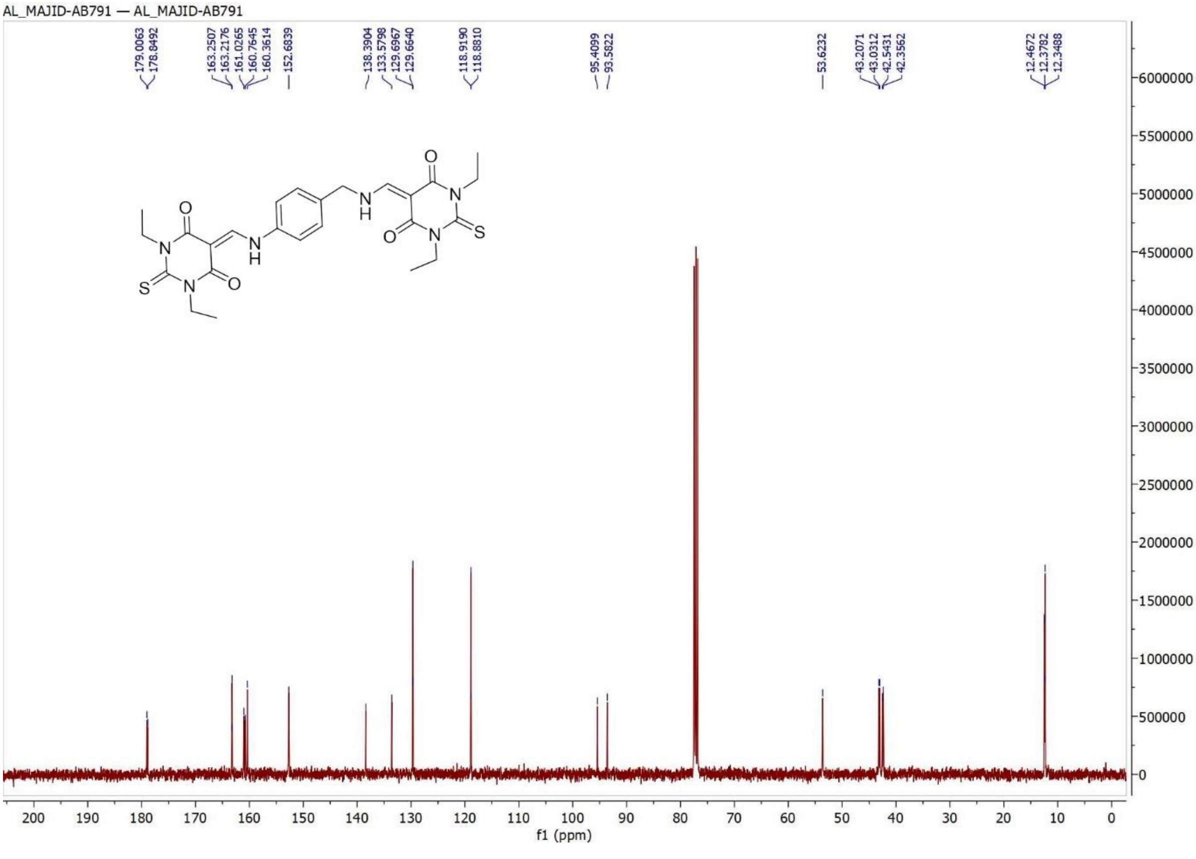

Supplement: Supplemental Material [file IENZ_A_1737045_SM4422.pdf]
